# Organizational Tensions in the Implementation of Modifiable Off-the-Shelf Technologies in a University Hospital: Qualitative Multimethod Study

**DOI:** 10.2196/84841

**Published:** 2026-05-13

**Authors:** Alessia Nowak, Carolina Gaul, Elena Hinz, Christine Knoll, Begüm Kül, Anja Wels, Julia C Berkmann, Cathrin de Pasquale, Gloria Kremser, Daniel Fürstenau, Akira-Sebastian Poncette, Felix Balzer, Lina Mosch

**Affiliations:** 1 Charité – Universitätsmedizin Berlin, Institute of Medical Informatics Berlin Germany; 2 Digital Health-Non-communicable Diseases University of Potsdam Hasso Plattner Institute Potsdam, Brandenburg Germany; 3 Charité – Universitätsmedizin Berlin, Business Unit Project Management Berlin Germany; 4 50Hertz Transmission GmbH, IT Portfolio Management, Digitalization Transformation Berlin Germany; 5 Charité – Universitätsmedizin Berlin, Business Unit IT, Back Office Berlin Germany; 6 School of Business & Economics, Freie Universität Berlin Berlin Germany; 7 Charité – Universitätsmedizin Berlin, Chief Medical Information Office Berlin Germany; 8 Charité – Universitätsmedizin Berlin, Department of Anesthesiology and Intensive Care Medicine Berlin Germany; 9 Berlin Institute of Health at Charité – Universitätsmedizin Berlin, BIH Biomedical Innovation Academy, BIH Charité Digital Clinician Scientist Program Berlin Germany

**Keywords:** health information technology, digital health, organization, qualitative research, implementation, tensions, paradoxes, user involvement

## Abstract

**Background:**

Hospitals face increasing pressure to accelerate digital transformation. Modifiable off-the-shelf technologies (MOTs) combine standardized products with limited adaptability, offering promising opportunities for rapid digitalization. However, implementing MOTs in complex hospital settings involves multiple barriers, facilitators, and organizational dynamics that require deeper investigation.

**Objective:**

This study identifies barriers and facilitators in the implementation of MOTs in hospitals and explores how organizational dynamics, conceptualized as tensions, emerge throughout this process.

**Methods:**

Guided by a constructivist-interpretivist paradigm, the study followed a collaborative action research approach. A qualitative multimethod design was used, including observations, workshops, and focus groups with clinical and project management staff. Data were analyzed using deductive-inductive qualitative content analysis following Kuckartz’s approach.

**Results:**

We analyzed 12 deidentified researcher protocols from 5 observations, 3 workshops, and 4 focus groups. Across these activities, 129 individuals from various wards and departments participated. Barriers and facilitators were clustered into 6 categories representing inhibiting and enabling conditions of MOT implementation. Barriers included product limitations (22.6%, 88/390), misaligned implementation process (4.4%, 17/390), absence of available individuals (3.8%, 15/390), structural challenges (2.8%, 11/390), and resource constraints (2.8%, 11/390). Facilitators included orchestrated implementation process (13.8%, 54/390), product alignment (14.4%, 56/390), effective coordination and communication (9.2%, 36/390), presence of available individuals (6.7%, 26/390), available resources (3.1%, 12/390), and structural assets (1.8%, 7/390). These categories were conceptualized into 3 organizational tensions: limited relative advantage of generic systems, structured participation challenges in complex settings, and constrained engagement despite motivation due to limited resources.

**Conclusions:**

Tensions in MOT implementation reflect organizational misfits that go beyond concrete barriers or facilitators and instead require systemic and agile organizational structures. The analysis introduces the concept of user:ability as an organizational capacity for enabling engagement with MOTs. We derive 3 tailored strategies to mitigate the tensions: ensuring relative advantage through IT integration and learning; empowering user participation through digital transformation and communication; and enabling clinician engagement through dedicated resources and systematic implementation. This study advances prior work by deepening the understanding of implementation determinants into organizational tensions and offering actionable strategies to address them. It also illustrates how collaborative, agile research approaches can illuminate complex organizational dynamics and support hospitals during transformative change.

## Introduction

A growing body of literature in information systems, health services, and implementation science highlights the potential of digital health technologies (DHTs) to improve health care delivery and outcomes [1-4]. Empirical studies demonstrate that DHTs, such as electronic medication systems, can enhance patient safety, care coordination, and clinical workflow efficiency [5-10].

In Germany, this recognition has been reinforced by major political initiatives. The Krankenhauszukunftsgesetz (Hospital Future Act; KHZG) created a strong regulatory impetus by tying substantial federal and state funding to the adoption of specific digital infrastructures [11]. Intended to improve care quality and efficiency, the KHZG mandates core digital solutions for German hospitals and penalizes nonimplementation with reductions of up to 2% of inpatient reimbursement, thereby enforcing rapid adoption [12,13]. Yet, while the KHZG accelerates digital adoption, DHTs remain subject to stringent safety and performance regulations, which can inadvertently slow innovation, delay responsiveness to emerging user needs, and limit design flexibility [14,15].

In this context, we conceptualize the DHTs examined in this study as modifiable off-the-shelf technologies (MOTs), drawing on modifiable off-the-shelf software or packaged software [16,17]. MOTs are prebuilt, licensed products that allow limited customization but are not developed in-house for a specific organizational use case. In this study, MOTs encompass a range of implementations: from a completed end user–initiated development scaled into the clinic, to system-wide infrastructures, and Software-as-a-Medical-Device, such as digital medication support systems [18], to applications closely integrated with hardware components, such as mobile ward rounds with iPads (Apple Inc). While MOTs promise rapid deployment and regulatory compliance, their limited adaptability often constrains alignment with heterogeneous clinical workflows and evolving organizational needs [19,20]. These dynamics can foster resistance and undermine organizational fit [20,21], particularly in large academic hospitals, where complexity heightens the risk of “unbridled adoption” [22]. In this context, the generic design of MOTs, developed for broad markets rather than the situated needs of specific clinical practices, often reduces usability because it lacks sensitivity to local workflows and limits opportunities for contextualized, implementation-level design [23]. As a result, successful implementation requires a stronger focus on end users during and after implementation, ensuring that users can use the system safely and efficiently through adequate training and sustained support [24].

Implementation science offers structured approaches to analyze these dynamics by examining the conditions under which adoption succeeds or fails. At its core is the examination of barriers and facilitators: barriers are factors that hinder or prevent uptake, while facilitators are enabling conditions within a specific context that promote adoption [25].

Previous research has identified barriers at multiple levels, including technological barriers such as interoperability [26,27] and system complexity [27,28]; individual-level barriers such as limited digital literacy [29] and low acceptance [29,30]; organizational barriers such as the lack of integration of digital technologies into existing workflows [30,31] and limited resources [29,31]; as well as structural barriers, including the lack of stakeholder involvement [26] and insufficient funding [32].

Beyond well-documented barriers, recent research points to deeper organizational dynamics, such as tensions and paradoxes, that are not easily resolved and require ongoing management [33]. Examples include conflicts between the push for standardization and the need for clinical autonomy; the pursuit of digital efficiency and the value of hands-on care; and the promise of technology and the reality of added burden, reflecting organization-technology misfits [33-35]. Building on Strong and Volkoff [19], misfits arise when technology and organizational practices fail to align. They appear in 6 domains: functionality, data, usability, role, control, and organizational culture. Each can surface either as a deficiency (missing features or capabilities) or as an imposition (an inherent technological characteristic needed for seamless integration) [19]. If left unmanaged, organization-technology misfits can impede adoption by fostering resistance, neglect, or misuse of otherwise beneficial technologies [33-35].

To address barriers, tensions, and misfits, implementation science applies evidence-based theoretical models and frameworks [21,36-40]. However, these are often insufficiently applied in practice [41], contributing to a persistent knowledge translation gap [42]. Action research can help bridge this gap by generating insights that are simultaneously actionable for organizations and generalizable for theory building [43]. Tailored implementation strategies [44] remain essential, as MOT implementation must account for technical and organizational factors alongside competing demands from regulations, limited resources, and staff needs.

Building on the outlined challenges and literature, we posit a set of assumptions that guide our study. As MOTs offer limited opportunities for participation in design, we expect usability and functionality misfits to emerge during implementation. Building on this premise, we suggest that effective MOT implementation requires tailored strategies to address contextual needs during implementation, as the limited modifiability of MOTs restricts contextual adaptation. We further argue that structural characteristics of hospitals can reinforce these misfits and intensify tensions during implementation.

These propositions lead to the following research question: What are the barriers and facilitators in the implementation of MOTs, and what organizational dynamics emerge during this process within complex hospital contexts?

## Methods

### Study Design

This study followed a qualitative, multimethod, collaborative participatory action research design within a constructivist-interpretivist paradigm. The design aimed to explore how clinical and project management staff experienced the implementation of MOTs in a complex tertiary hospital environment [45,46]. Action research was conducted in iterative Plan-Act-Observe-Reflect cycles [47,48], integrating observations, focus groups, and participatory workshops in a convergent triangulation design. Following the action research paradigm, female implementation researchers with backgrounds in clinical medicine, human factors, and information systems acted as the action researchers, whereas female members of the Chief Medical Information Office and the operational project management unit represented the client system. Participants’ accounts were treated as first-order data and interpreted by the researchers to develop second-order concepts [49]. As some team members were involved in coordinating implementation activities, their dual roles not only provided valuable contextual insight but also introduced the possibility of bias. Reflexivity was supported through systematic field notes, joint data collection, and regular team discussions, and analytic distance was strengthened by involving 2 researchers (BK and DF), who had not participated in data collection, in leading the analysis. The study was reported in accordance with the Standards for Reporting Qualitative Research (SRQR; see Multimedia Appendix 1) and the Consolidated Criteria for Reporting Qualitative Research (COREQ; see Multimedia Appendix 2) [50,51].

Before data collection, participants were informed about the study’s purpose, the researchers’ roles, and the goals of the initiative. No formal relationships were established in advance. Researchers were introduced in line with their collaborative roles as action researchers (AN, CG, EH, and LM) and clients (AW, CdP, JCB, and GK) [47]. Professional backgrounds and interest in implementation processes were disclosed, whereas the research questions were not shared.

### Setting

The study was conducted at Charité – Universitätsmedizin Berlin, a large German university hospital employing over 23,000 staff and operating more than 3000 beds across more than 100 clinics and departments [52].

The research was embedded in the hospital’s digital transformation initiative funded under the German Hospital Future Act (KHZG), which promotes the implementation of DHTs across German hospitals.

**Figure 1:**
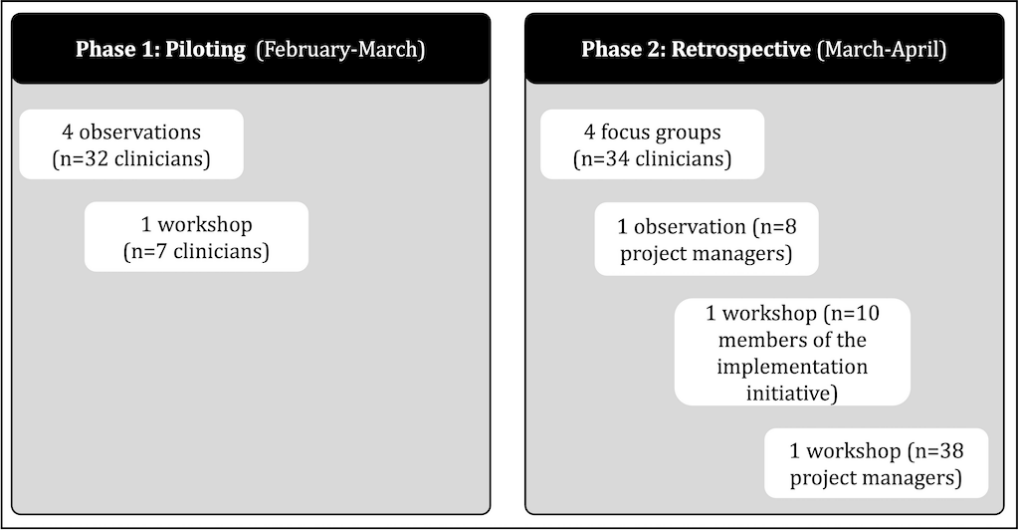
Data collection procedure for a qualitative study on modifiable off-the-shelf technology implementation at a large German academic hospital, illustrating a 2-stage process (pilot and evaluation phases) conducted from February to April 2025 across clinical and administrative settings.

Recruitment and data collection occurred from February to April 2025 across 2 implementation phases: (1) a pilot phase observing pre- and early implementation activities and (2) an evaluation phase retrospectively assessing experiences across wards and departments. Together, these stages provided rich insights into the implementation process (Figure 1). Data were collected in inpatient wards, emergency and outpatient clinics, and administrative project management offices.

Access to the field was established by the initiative’s lead via email, drawing on existing professional relationships to promote trust and encourage engagement, as well as through presentations of the initiative in various internal organizational committees. To contextualize these implementation activities, the next section describes the portfolio of digital technologies introduced under the KHZG initiative and examined in this study.

### The Implementation Portfolio

The research was conducted within the framework of the Hospital Future Act, a political mandate that allocates targeted funding to accelerate hospitals’ digital transformation. Hospitals apply for these funds and must comply with national standards for DHTs to be eligible for support [11]. In line with the requirements of the KHZG and the hospital’s overarching digital strategy [53], which emphasizes interoperability, user-centered design, and improved care, a dedicated implementation initiative was launched. Its objective was to introduce 10 preselected MOTs that fulfill KHZG criteria and contribute to the operationalization of the telematics infrastructure, Germany’s secure and interoperable digital health network [54]. These technologies, formally approved as part of the hospital’s digital transformation program, were implemented across inpatient and outpatient departments to enhance clinical documentation, resource management, device integration, and interprofessional communication. Patient-facing or consumer-oriented applications were not included because they were not part of the active implementation portfolio during the study period and thus did not constitute the exposure of interest. The study aimed to investigate the experiences, practices, and challenges of clinical personnel as active users of KHZG-funded MOTs, rather than to evaluate patient experiences or patient-technology interactions. The portfolio comprised both mandatory and optional solutions (see Table 1). Aligning with the action research approach [47], the initiative was iteratively refined in response to contextual factors, including high workloads and limited resources, as well as ongoing adjustments to evolving KHZG regulations.

**Table 1:** Overview of mandatory and optional modifiable off-the-shelf technologies included in the implementation portfolio of a digital transformation initiative at a large German university hospital (February-April 2025). Technologies were implemented in inpatient and outpatient clinical areas as part of the KHZG^a^-funded program and represent the digital tools to which clinical staff were exposed during the study.

Technology	Description	Requirement type
Electronic Sick Leave Certificate [55]	Online transmission of sick leave certificates via the telematics infrastructure.	Mandatory
Nationwide electronic health record (elektronische Patientenakte) [56]	Integration and implementation of a unified IT system for medical report writing across the hospital.	Mandatory
ePrescription Implementation [57]	Digital creation and transmission of prescriptions via the telematics infrastructure.	Mandatory
Digital Medication Management (inpatient) [58]	Digital documentation of medication on regular wards and some outpatient clinics; work on emergency and intensive care units in progress.	Mandatory
KIM (Communication in Medicine) – KIM Email [59]	Secure, encrypted email service within the telematics infrastructure for medical communication.	Optional
Operating Room Solutions (Digital Implant Passport) [60]^b^	Clinical information system–integrated software module for implant documentation.	Optional
Mobile Ward Round [61]	An iPad app allowing access to critical clinical systems/features to support clinical ward rounds for doctors, nurses, and other staff (Wi-Fi dependent).	Optional
Admission Planning Tool [62]^b^	Standardized planning for inpatient admissions.	Optional
Fluid Balance Module [63]^b^	Digital documentation of access points, drains, and fluid balances in the clinical information system.	Optional
Digital Whiteboard for Ward Management [64]^c^	Digital workflow organization tool for inpatient services in hospital wards and intensive care units.	Optional

^a^KHZG: Krankenhauszukunftsgesetz (Hospital Future Act).

^b^SAP modules were modified by the local IT department.

^c^End user–initiated development.

### Participants

Participants were clinical staff (physicians, nurses, and patient coordinators) and nonclinical project management personnel involved in or affected by the digitalization initiative.

Eligibility criteria were (1) employment at Charité in a clinical or project management role and (2) involvement in or exposure to at least one MOT implementation. Recruitment followed a multistage purposeful sampling strategy to ensure maximum variation across disciplines and hierarchical levels [65]:

- Stage 1 (maximum variation sampling): selection of diverse departments (regular ward, emergency, and outpatient settings).
- Stage 2 (emergent purposive sampling): inclusion of additional participants expressing interest during project meetings and presentations.
- Stage 3 (criterion sampling): targeted recruitment of project managers due to their pivotal role in planning and coordination.

Elements of convenience sampling were used when data collection was integrated into existing meeting schedules.

A total of 129 participants were included: 73 clinicians, 46 project managers, and 10 members of the implementation initiative. While participation was generally high, a small number of invited nurses and physicians declined due to time constraints. Exact numbers of refusals and dropouts were not systematically recorded, as participation was integrated into routine clinical and project management activities. Sampling decisions across all stages aimed to capture diverse perspectives from frontline users and institutional implementers. Units of study were 12 researcher protocols: 5 from observations, 4 from focus groups, and 3 from workshops (see Multimedia Appendix 3). While 129 individuals contributed to data collection activities, analytic inferences were drawn from these protocols rather than from individual participants.

Demographic data were not collected because, following Rapoport’s [43] view of action research as a collaborative, context-bound process focused on collective practice change, roles and functions in implementation were considered more relevant than individual characteristics, and statistical generalizability was not sought.

### Assessments

Primary outcomes were perceived barriers and facilitators to MOT implementation, as well as underlying organizational dynamics that became evident during the process.

Exposures of interest included the characteristics of MOTs (usability, adaptability, and interoperability) and organizational factors (resources, infrastructure, and communication structures).

Potential contextual influences considered during interpretation included differences in departmental resources, professional hierarchies, and workload variation.

All outcomes were derived from qualitative accounts rather than standardized instruments, reflecting participants’ lived experiences and professional perspectives.

### Data Sources

#### Overview

An emergent qualitative design [66] guided data collection. Outcomes were assessed using 3 qualitative data sources: focus groups [67], workshops [68], and field observations [69] (see Multimedia Appendix 3). Data collection was conducted across 2 implementation phases and combined real-time engagement with retrospective reflection to capture both situated practices and reflective accounts of barriers, facilitators, and organizational dynamics. Each method contributed distinct perspectives on implementation processes and context-specific determinants.

#### Field Observations (n=5 Protocols)

Field observations were conducted on-site or online to capture real-time implementation practices, clinical routines, and interactional dynamics within the organizational environment. In phase 1, researchers (AN, CG, LM, AW, JCB, GK, and CdP) conducted 4 departmental observations (each 60-90 minutes; protocols [P] 9-12), focusing on implementation workflows and situated engagement with available MOTs, including how interest in specific solutions emerged or failed to emerge. In phase 2, a 90-minute online observation with project managers provided broader perspectives on cross-departmental coordination processes (P2). Researchers adopted a participant-observer stance, with varying levels of engagement depending on the activity. Observational field notes documented contextual factors, actor interactions, emergent issues, and workflow characteristics [69]. Field observations focused on capturing situated practices and real-time interactions as they occurred in the clinical and organizational context, rather than participants’ retrospective accounts or evaluative judgments.

#### Workshops (n=3 Protocols)

Workshops served as participatory formats to support collective reflection and cocreate implementation strategies. In phase 1, following a ward’s expressed interest in an MOT, AN and EH conducted a 2-hour back-casting workshop [70] with senior and assistant physicians, nursing staff, and patient coordinators to envision future workflows and articulate required process changes, starting from a desired future state (P4). In phase 2, a 30-minute workshop with project managers (P6), conducted by CG, AN, and EH, focused on evaluating implementation steps (“do,” “don’t,” “must”) and strategies for resource-constrained contexts. A 1-hour internal workshop with implementation initiative members (P7), led by CG and AN, supported reflexive examination of the overall approach. Workshops followed a semistructured but flexible facilitation style to allow participant-led exploration of barriers, facilitators, and implementation needs. They were explicitly future-oriented and designed to support joint sense-making, cocreation, and the development of shared implementation strategies.

#### Focus Groups (n=4 Protocols)

In phase 2, CG, AN, JCB, CdP, and AW conducted 4 one-hour focus groups with clinicians across diverse professional and hierarchical levels (P1, P3, P5, and P8). Focus groups explored implementation experiences, communication preferences, perceived determinants of adoption, and emergent best practices. Sessions were held on-site or digitally and were researcher-moderated. Focus groups elicited retrospective reflections, complementing the observational insights generated in phase 1. In contrast to workshops and observations, focus groups emphasized retrospective reflection on implementation experiences and facilitated comparison of perspectives across professional roles.

Across all methods, manual note-taking (without audio or video recordings) ensured participant comfort, protected confidentiality, and supported candid dialogue. A structured field note template (see Multimedia Appendix 4) was developed specifically for the study to document setting characteristics, actor interactions, contextual influences, and emerging themes consistently across methods. Only study participants and researchers were present during data collection; no nonparticipating individuals attended. Field notes were primarily compiled by AN and CG and supplemented by LM, CdP, AW, and JCB, enabling iterative refinement of prompts and observational foci in response to field engagement [66].

### Study Size

The final study size (n=129 participants across 12 protocols) was determined pragmatically, based on data sufficiency, diversity of perspectives, and information power. This approach aligns with qualitative research conventions rather than statistical power considerations [71]. Breadth and representativeness across roles and departments were prioritized over saturation, given the multimethod, organizational scope of the research.

### Data Analysis

**All analyses were conducted at the protocol level, with the** 12 researcher protocols forming the analytic corpus. Coding frequencies indicate patterns across activities rather than individual-level quantification, consistent with the study’s action research design [47].

All protocols were transcribed and analyzed in MAXQDA 2022 (VERBI GmbH) [72] using deductive-inductive qualitative content analysis following Kuckartz and Rädiker [73].

To ensure analytic rigor, all coding activities were conducted by product-independent action researchers who were not part of the client system. AN and CG defined a priori codes from the Consolidated Framework for Implementation Research [21,36], guided by prior knowledge of barriers and facilitators. An independent researcher (BK), who was not involved in data collection, then coded the material in MAXQDA, applying these codes and developing inductive ones specific to the MOT context. BK subsequently assigned these codes to either the first-order category MOT implementation barrier or MOT implementation facilitator, with regular review by AN and CG. AN grouped related codes into 12 second-order categories (6 barriers and 6 facilitators), which were validated in 3 team meetings (AN, CG, and LM). To prevent “data overload,” we decided to report only codes with 4 or more coded segments [74]. As Kuckartz and Rädiker [73] do not provide numerical thresholds, these cutoffs were established through analytical reflection to manage data complexity and ensure transparent reporting. This approach aligns with qualitative standards that emphasize conceptual significance over statistical representativeness. A detailed overview of coding frequencies is provided in Multimedia Appendix 5.

Building on preliminary qualitative findings from the different data collection steps, MOT implementation barriers and facilitators also served as a basis for analyzing organizational tensions and deep-seated dynamics. Together, the 3 data sources provided complementary perspectives on the implementation of MOTs. Field observations captured how available MOTs were encountered in practice and how reasons for adoption or nonadoption emerged in everyday clinical and organizational workflows. Workshops contributed to the structured, future-oriented articulation of implementation pathways, including required process changes and strategies for resource-constrained contexts. Focus groups supported retrospective reflection on individual implementation experiences, preferences, and perceived determinants of adoption. Additionally, findings from the different data collection steps were triangulated to inform the interpretation of recurring patterns and contradictions. Based on access to the coded material, LM and DF, who were not involved in data collection and therefore contributed additional analytical distance, independently examined codes for contradictory demands or competing logics (eg, complexity vs participation, resource constraints vs engagement). Findings were discussed in 3 team meetings with AN and CG.

Integrating findings across methods, perspectives, and study phases, the analysis served as the basis for identifying higher-order organizational tensions. As a result, the final conceptual interpretations and tailored implementation strategies were derived from this integrated, triangulated analytical process.

Triangulation of methods (observations, workshops, and focus groups) and perspectives (clinicians, project managers, and implementation staff) enhanced trustworthiness [75]. Reflexivity was maintained through analytic memos and peer debriefing sessions. Quantitative counting of coded segments (frequencies and percentages) was used descriptively to illustrate emphasis, not to infer significance.

### Ethical Considerations

#### Ethics Approval

The study protocol was reviewed and approved by the Charité – Universitätsmedizin Berlin Ethics Committee (approval number EA4/031/23). As the study involved human participants in the form of clinical and project management staff, institutional ethics oversight was required and obtained.

#### Informed Consent

Before participation, all individuals received verbal information about the study’s aims, procedures, voluntary nature, and their right to withdraw at any time without consequences. Verbal informed consent was obtained before all data collection activities. The ethics committee confirmed that verbal consent was appropriate given the minimal risk, nonclinical nature of the study, and the absence of patient-related data.

#### Privacy and Confidentiality

To protect participant confidentiality, all field notes and workshop and focus group documentation were deidentified at the point of data collection. No names, professional identification numbers, or other personal identifiers were recorded. Data were stored on secure, password-protected institutional servers accessible only to the research team. Findings are presented exclusively in aggregated or anonymized form.

#### Compensation

Participants did not receive any financial or material compensation for their involvement. Participation was voluntary and occurred during or adjacent to routine professional activities.

#### Identifiability in Images or Multimedia Appendices

No identifiable individuals are presented in the manuscript or its multimedia appendices. All illustrative figures and multimedia appendices contain only anonymized documents and deidentified field note excerpts. No visual materials include recognizable individuals; therefore, no individual image-based consent forms were required.

## Results

### Overview and Analytical Structure

**Figure 2:**
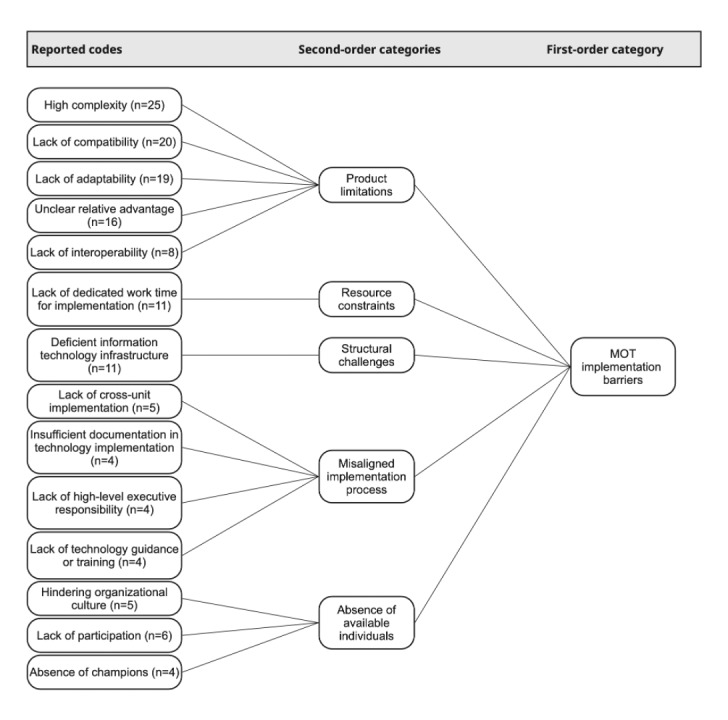
Coding scheme of modifiable off-the-shelf technology (MOT) implementation barriers. Reported codes were aggregated into 5 second-order categories—product limitations, resource constraints, structural challenges, misaligned implementation processes, and absence of available individuals—which together constitute the overarching category of MOT implementation barriers.

**Figure 3:**
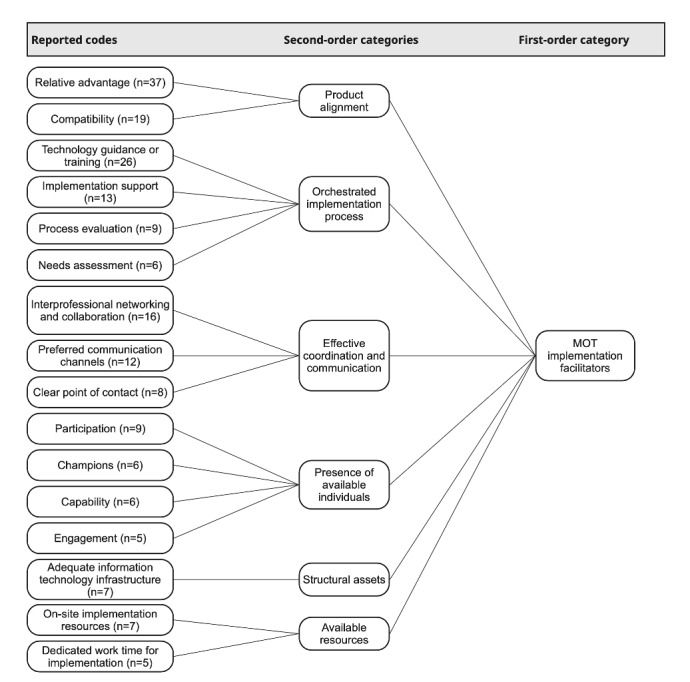
Coding scheme of modifiable off-the-shelf technology (MOT) implementation facilitators. Reported codes were grouped into 6 second-order categories—product alignment, available resources, structural assets, an orchestrated implementation process, the presence of available individuals, and effective coordination and communication—which together constitute the overarching category of MOT implementation facilitators.

Results are presented in 2 sections: (1) 6 integrated second-order categories combining barriers and facilitators within shared aspects of MOT implementation (see Figures 2 and 3) and (2) organizational dynamics conceptualized as 3 tensions. We defined barriers as factors that hinder or prevent uptake and facilitators as enabling conditions that support uptake [25]. Codes and second-order categories were labeled according to participants’ perceptions and our interpretation: barriers were typically described using terms such as lack of, limited, or deficient, whereas facilitators were usually characterized by the perceived presence or availability of a factor.

In total, the coding process identified 390 data segments, comprising 172 barriers and 218 facilitators across the 12 researcher protocols. Frequencies indicate how often themes occurred across protocols and are not intended to represent participant-level prevalence. Multimedia Appendix 5 provides an overview of the distribution of reported and unreported codes, as well as all code frequencies. Representative quotes for all codes can be found in Multimedia Appendix 6. The following sections elaborate on each second-order category, beginning with those related to product characteristics.

### Product Limitations and Product Alignment

This category captures how the functional, technical, and workflow-related characteristics of MOTs shaped users’ experiences.

Barriers arose when MOTs were difficult to operate, poorly aligned with existing routines, or technically constrained. These challenges were reflected in the lack of compatibility (20/172, 11.6%, barriers; 20/390, 5.1%, coded segments), such as when duplicate documentation or parallel paper processes were required. Lack of adaptability (19/172, 11%, barriers; 19/390, 4.9%, coded segments) captured the limited flexibility of MOTs to accommodate diverse wards, workflows, or professional roles. The added value of MOTs was not always evident, as reflected in unclear relative advantage (16/172, 9.3%, barriers; 16/390, 4.1%, coded segments). Difficulties were further reinforced by missing interfaces and fragmented data flows, represented by a lack of interoperability (8/172, 4.7%, barriers; 8/390, 2.1%, coded segments), which required manual bridging and complicated coordination across units. High complexity (25/172, 14.5%, barriers; 25/390, 6.4%, coded segments) referred to multistep logins, unstable Wi-Fi, and slow system responses that conflicted with fast-paced clinical work, as exemplified by a participant describing the mobile ward round:

[Mobile Ward Round] You go into the room to see the first patient, document everything, then go to the second patient, then the iPad is off, you have to enter a code and unlock it again, then log back into the HIS – by the time you've logged in, 3-5 minutes have passed and the others are already with the next patient. Then you want to look at the MRI image again, but the Wi-Fi cuts out. So, the idea is great, but the application isn’t

P9

Facilitators emerged when MOTs aligned with clinical routines and offered clear benefits. Relative advantage was the most frequently reported facilitator (37/218, 17%, facilitators; 37/390, 9.5%, coded segments), emphasizing efficiency gains and reduced communication effort. Compatibility (19/218, 8.7%, facilitators; 19/390, 4.9%, coded segments) supported adoption when system functions fit existing workflows or professional expectations.

Together, these patterns indicate that MOT uptake depended on the extent to which technical design, usability, and workflow integration cohered with everyday clinical practice.

### Resource Constraints and Available Resources

This category captures how staffing levels, protected time, and available support shaped clinicians’ ability to engage in MOT implementation activities.

The most prominent barrier was a lack of dedicated work time for implementation, which accounted for 11 out of 172 (6.4%) barriers and 11 out of 390 (2.8%) coded segments. Participants reported that implementation-related tasks had to be completed alongside routine clinical duties, leaving little opportunity for training, experimentation, or supporting colleagues. As P7 noted: “Enthusiasm is there, but the time resource is missing.”

Facilitators emerged when additional resources or protected time were available. On-site implementation resources represented 7 out of 218 (3.2%) facilitators and 7 out of 390 (1.8%) coded segments, and dedicated work time for implementation accounted for 5 out of 218 (2.3%) facilitators and 5 out of 390 (1.3%) coded segments. Participants described that responsive local support and focused time windows enabled them to engage in implementation activities without competing clinical responsibilities and to incorporate MOTs more consistently into everyday practice.

### Structural Challenges and Structural Assets

This category reflects how participants experienced the adequacy and reliability of the hospital’s technical infrastructure during MOT use and implementation.

Barriers were primarily captured in the code-deficient IT infrastructure, which accounted for 11 out of 172 (6.4%) barriers and 11 out of 390 (2.8%) coded segments. Participants described limited device availability, unstable Wi-Fi, slow system performance, and outdated hardware, all of which interfered with routine work and discouraged sustained use of MOT functions. Furthermore, these gaps sometimes prevented MOTs from being implemented in the first place, as required equipment was delayed or unavailable. As one participant noted: “Procurement of technical infrastructure unclear or very slow and bureaucratic via procurement platform” [P3].

By contrast, facilitators emerged when the infrastructure functioned reliably. Adequate IT infrastructure accounted for 7 out of 218 (3.2%) facilitators and 7 out of 390 (1.8%) coded segments. Participants reported smoother logins, stable network access, and consistent availability of clinical information; these conditions supported the routine use of MOTs and reduced the need for workaround practices.

### Misaligned Implementation Process and Orchestrated Implementation Process

This category captures how the structure, coordination, and support of implementation activities influenced clinicians’ experiences.

Barriers reflected fragmented processes, unclear responsibilities, and insufficient preparation. Lack of cross-unit implementation (5/172, 2.9%, barriers; 5/390, 1.3%, coded segments) and insufficient documentation in technology implementation (4/172, 2.3%, barriers; 4/390, 1.0%, coded segments) highlighted difficulties in understanding which technologies were already in use and how previous rollouts had unfolded. Several participants reported that oversight for implementation had been delegated to local clinical leaders who, due to existing clinical demands, had limited capacity to take on additional coordination tasks, as reflected in the lack of high-level executive responsibility (4/172, 2.3%, barriers; 4/390, 1.0%, coded segments). Reports of lack of technology guidance or training (4/172, 2.3%, barriers; 4/390, 1.0%, coded segments) further contributed to inconsistent processes and limited practical support.

Facilitators emerged when implementation was structured and actively supported. Technology guidance or training was a frequent facilitator in this domain (26/218, 11.9%, facilitators; 26/390, 6.7%, coded segments), alongside implementation support (13/218, 6.0%, facilitators; 13/390, 3.3%, coded segments), process evaluation (9/218, 4.1%, facilitators; 9/390, 2.3%, coded segments), and needs assessment (6/218, 2.8%, facilitators; 6/390, 1.5%, coded segments). Participants emphasized that accessible, context-specific training formats, visible support from the implementation team, and iterative feedback loops helped align expectations and adapt strategies to local workflows: “Planned iterative approach from the start—reminding people, asking about further interaction” [P7].

### Absence of Available Individuals and Presence of Available Individuals

This category reflects how the availability, engagement, and roles of clinical staff and members of the implementation team were experienced by participants during MOT adoption.

Barriers were reported when individuals lacked time, recognition, or opportunity to participate meaningfully. Lack of participation accounted for 6 out of 172 (3.5%) barriers and 6 out of 390 (1.5%) coded segments. Participants described that, although interest often existed, competing clinical duties limited involvement. Hindering organizational culture represented 5 out of 172 (2.9%) barriers and 5 out of 390 (1.3%) coded segments, referring to hierarchical structures and strained working climates that reduced openness to change. The absence of champions accounted for 4 out of 172 (2.3%) barriers and 4 out of 390 (1.0%) coded segments, indicating missing local multipliers who could support peers or coordinate with the project team.

By contrast, facilitators emerged when individuals—whether clinicians or implementation team members—were available and engaged. Participation accounted for 9 out of 218 (4.1%) facilitators and 9 out of 390 (2.3%) coded segments, with staff actively initiating or supporting MOT rollout activities. Presence of champions represented 6 out of 218 (2.8%) facilitators and 6 out of 390 (1.5%) coded segments, capturing designated or informal multipliers who guided colleagues, tested functions, or liaised with the implementation team. Capability accounted for 6 out of 218 (2.8%) facilitators and 6 out of 390 (1.5%) coded segments, with participants highlighting digital competence and willingness to learn. Engagement accounted for 5 out of 218 (2.3%) facilitators and 5 out of 390 (1.3%) coded segments, reflected in proactive contributions to problem solving or workflow adaptation. A participant summarized the importance of identifiable and engaged individuals: “What else do we need from the clinics? Clear, consistent contacts who can serve as multipliers” [P6].

### Effective Coordination and Communication

This category captures how interprofessional coordination and communication practices shaped the implementation of MOTs.

Although participants occasionally referred to coordination challenges, no communication-related barrier reached the minimum reporting threshold of 4 coded segments used in this study. Facilitators, however, were more frequently described and were reflected in interprofessional networking and collaboration, preferred communication channels, and a clear point of contact.

Interprofessional networking and collaboration accounted for 16 out of 218 (7.3%) facilitators and 16 out of 390 (4.1%) coded segments. Participants emphasized that structured collaboration between clinical professions and the project team supported implementation activities, as illustrated by one clinician who noted the value of “bringing different professional groups together to plan the implementation” [P6]. Preferred communication channels represented 12 out of 218 (5.5%) facilitators and 12 out of 390 (3.1%) coded segments, highlighting the importance of using familiar and accessible communication formats, such as in-person briefings, emails, or departmental meetings. A clear point of contact accounted for 8 out of 218 (3.7%) facilitators and 8 out of 390 (2.1%) coded segments, emphasizing the role of identifiable, reliable contacts for resolving questions and maintaining continuity during implementation.

### Tensions in MOT Implementation Resulting From Existent and Perceived Barriers and Facilitators

#### Organizational Tensions in MOT Implementation

**Figure 4:**
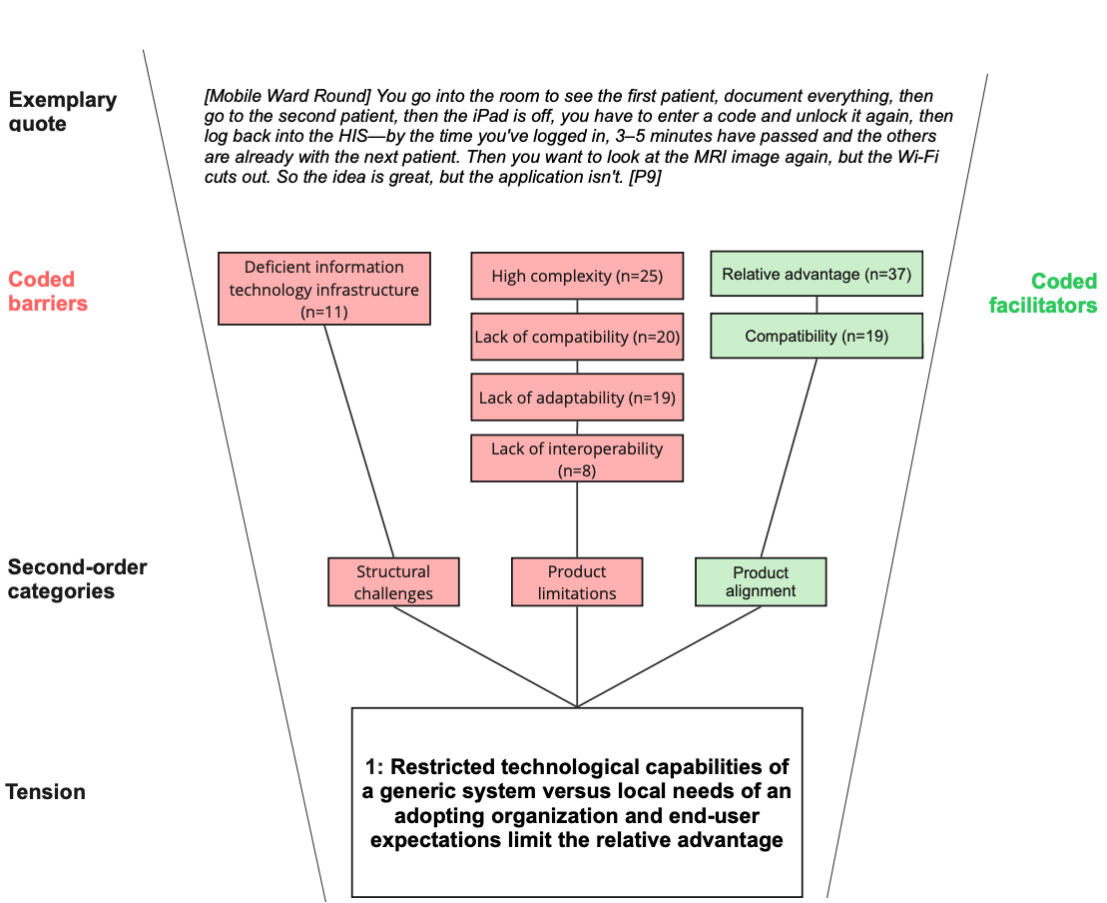
Coding structure illustrating barriers and facilitators associated with tension 1 (generic system capabilities vs local clinical needs) in the implementation of modifiable off-the-shelf technologies at a large German academic hospital. Reported codes were aggregated into second-order categories that together capture the tension between standardized system design and the requirements of local clinical workflows. Exemplary quotes illustrate the underlying dynamics.

**Figure 5:**
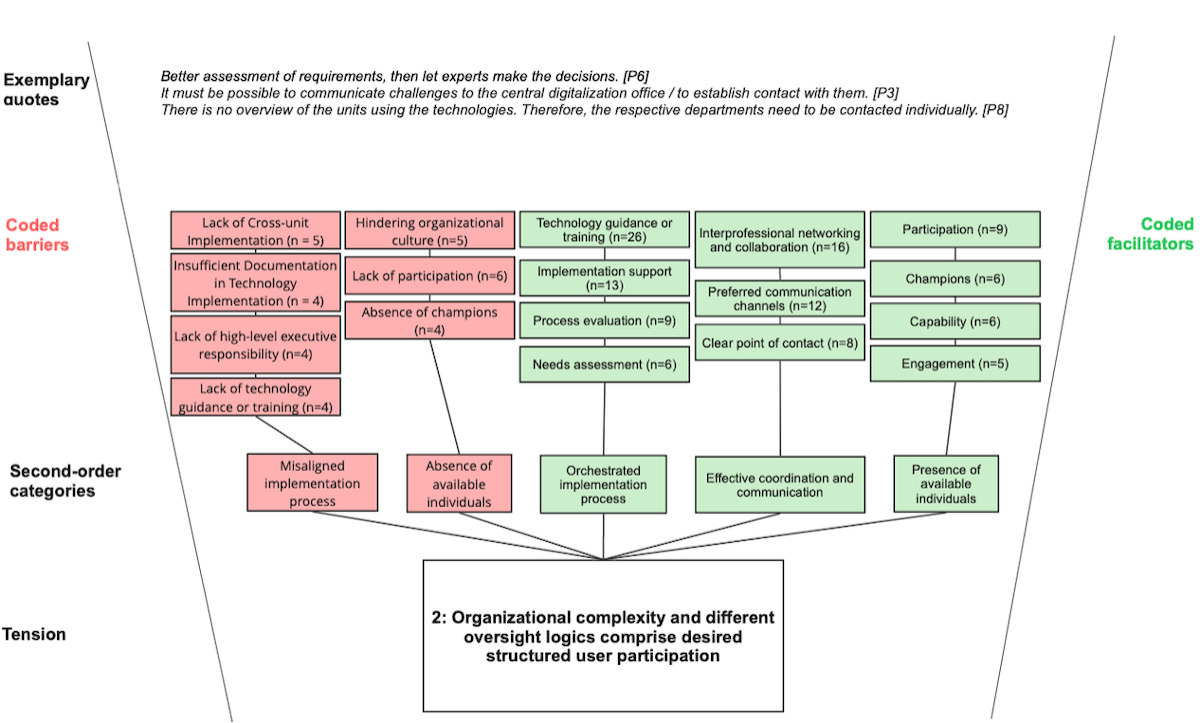
Coding structure illustrating barriers and facilitators associated with tension 2 (organizational complexity vs structured user participation) in the implementation of modifiable off-the-shelf technologies at a large German academic hospital. Reported codes were aggregated into second-order categories that together capture the tension between organizational oversight structures and desired user participation. Exemplary quotes illustrate the underlying dynamics.

**Figure 6:**
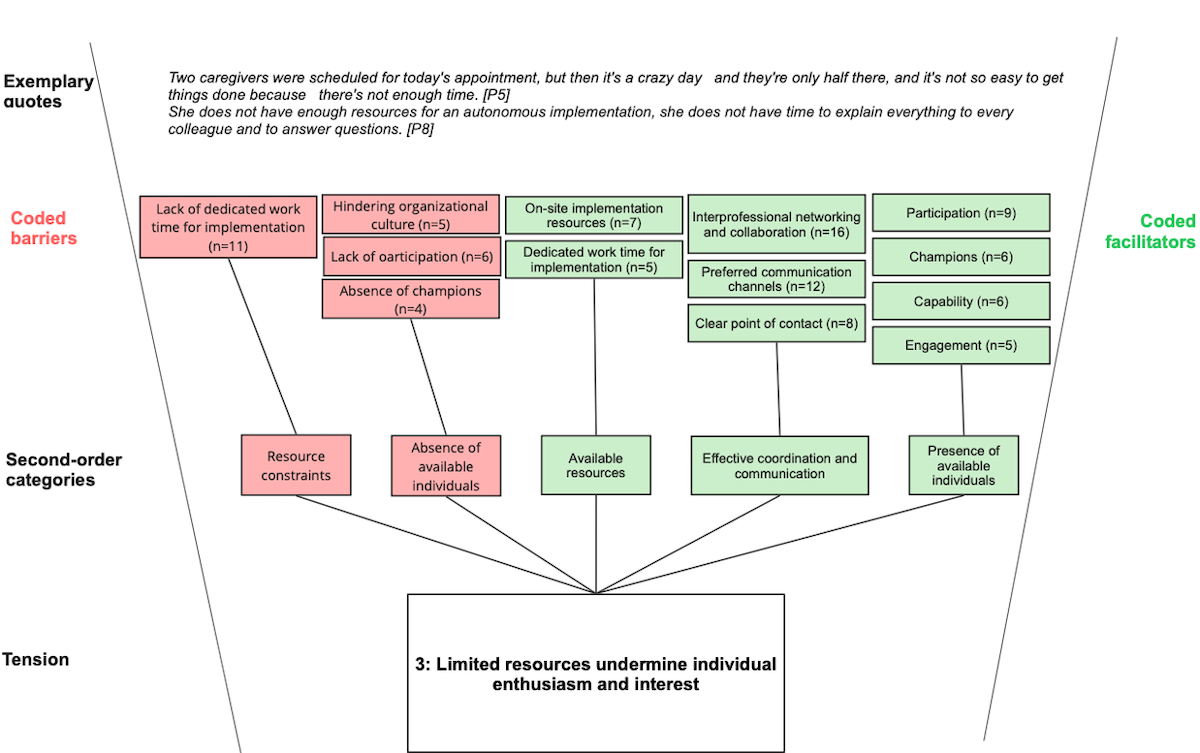
Coding structure illustrating barriers and facilitators associated with tension 3 (limited resources vs individual enthusiasm) in the implementation of modifiable off-the-shelf technologies at a large German academic hospital. Reported codes were aggregated into second-order categories that together capture the tension between constrained organizational resources and the motivation of clinicians and project teams to support implementation. Exemplary quotes illustrate the underlying dynamics.

Across all categories, our analysis revealed deeper, interrelated patterns beyond isolated barriers or facilitators, pointing to underlying organizational tensions that become apparent during MOT implementation and use. We identified 3 organizational tensions that characterize MOT implementation in complex academic hospital settings (see Figures 4-6).

#### Tension 1: Restricted Technological Capabilities of a Generic System Versus Local Needs of an Adopting Organization and End User Expectations Limit The Relative Advantage

While users expected a relative advantage from MOT use, product limitations emerged as a major barrier. Lack of interoperability and lack of compatibility led to fragmented data flows and incomplete functional integration, as vendor-driven system designs using preconfigured, standardized interfaces conflicted with the locally required data exchange in everyday care, which is often based on organization-specific terminology and interfaces. This created a tension because standardized system logics prioritized general applicability across diverse settings, whereas local care practices required flexible, context-specific integration of data flows. As a result, users were confronted with constant translation and alignment work to bridge the gap between the generic system architecture and their actual needs.

Closely related, the lack of adaptability and high complexity of MOTs produced usability constraints, forcing workaround practices as MOT use collided with the temporal, relational, and situational logics of clinical practice. This stood in tension with the expectation that digital systems should simplify and streamline clinical work, as MOTs instead introduced additional layers of cognitive and procedural effort. In practice, clinicians had to spend more time navigating complex interfaces or bypassing rigid functions, which diverted attention from patient care.

The emerging technological tension was further exacerbated by structural challenges, another major barrier category, particularly the hospital’s deficient IT infrastructure. Ultimately, MOTs were not adopted when their potential efficiency and performance gains were outweighed by these barriers. These included high complexity, limited adaptability, and IT-infrastructural constraints, particularly a lack of interoperability and compatibility. Under such conditions, the relative advantage of MOTs was not apparent to users, which hindered their uptake.

#### Tension 2: Organizational Complexity and Different Oversight Logics Compromise Desired Structured User Participation

Clinicians emphasized the importance of active involvement before and during MOT implementation through preimplementation needs assessment, implementation support, process evaluation, and the use of preferred communication channels to share information effectively. Likewise, project managers highlighted their need for clear points of contact and designated champions for MOT implementation within departments. While participation was valued and desired by both groups, a hindering organizational culture was reported by clinicians and the implementation team. Clinicians, for example, felt that too much responsibility for MOT implementation was shifted to local clinical leaders, indicating a mismatch between assigned responsibilities and their perceived roles.

Participants perceived misaligned implementation processes—such as lack of technology guidance or training, insufficient documentation in technology implementation, and lack of cross-unit implementation—as barriers to their participation. These challenges may reflect differing oversight logics and governance structures shaped by managerial, economic, and regulatory requirements, such as the KHZG and data protection regulations, which had to be implemented within a short time frame, leaving limited opportunity for thorough planning and preparation. Specifically, rigid, project-bound management, hierarchical structures, and strained working climates reduced openness to participatory formats (eg, needs assessment) and hindered effective coordination and communication. This created a tension between the participatory processes that clinicians valued and the formal structures guiding implementation.

#### Tension 3: Limited Resources Undermine Individual Enthusiasm and Interest

While some participants expressed enthusiasm and willingness to participate, a major barrier to MOT implementation was resource constraints. Clinicians criticized the lack of dedicated work time they could allocate to implementation and highlighted their need for intensive on-site implementation support from project managers. Project managers, in turn, lacked champions in clinical departments for rollout processes and faced IT staff shortages and tight rollout schedules. This tension arises when clinicians are assigned responsibility as implementation leaders without having the necessary resources or identifying with that role. As a result, hospital managers experience tensions because they must operate under resource constraints while facing a persistent need for dedicated implementation resources.

Furthermore, interprofessional collaboration was recognized as a facilitator of MOT implementation because it was perceived to help align perspectives and create shared ownership. However, in practice, training and support were often organized in monoprofessional silos, meaning that doctors and nurses were trained separately. This reduced opportunities for joint learning and undermined the collaborative momentum required for sustained adoption. These structural and organizational barriers not only slowed implementation processes but also gradually weakened the initial enthusiasm of both clinicians and project managers. This reflects tensions between the demand to be innovative and collaborative and the lack of structures and resources needed to facilitate such collaboration.

## Discussion

### Principal Findings

This study identified 3 tensions during the implementation of MOTs.

Tension 1 concerned restricted technological capabilities that did not meet local needs and reduced the perceived relative advantage of the MOTs. Key barriers included product limitations, such as limited interoperability, low compatibility, low adaptability, and high complexity, as well as structural challenges that hindered smooth use. Product alignment acted as a facilitator when MOTs fit existing workflows.

Tension 2 arose from organizational complexity and differing oversight logics that compromised structured user participation. Barriers included a misaligned implementation process and the absence of available individuals to coordinate or support activities. Facilitators included an orchestrated implementation process, effective coordination and communication, and the presence of available individuals who could enable cross-unit participation.

Tension 3 reflected limited resources that undermined individual enthusiasm and interest in implementation. Barriers included resource constraints and a recurring absence of available individuals. Facilitators involved available resources, effective coordination and communication, and the presence of available individuals who could help maintain engagement.

The remainder of the “Discussion” section links these tensions and their respective barriers to misfit theory, introduces the concept of user:ability, and examines how 3 tailored strategies that integrate the observed facilitators respond to the organizational tensions in MOT implementation.

### Misfits and the Role of User:Ability

Across all 3 tensions, the findings reveal that hospitals operate in a policy environment that seeks to accelerate digitalization while imposing strict timelines and compliance demands. Examples include Germany’s KHZG and telematics infrastructure requirements [11,54], EU regulations such as General Data Protection Regulation (GDPR) [76] and the upcoming European Health Data Space [77], and US initiatives like HITECH [78] and the 21st Century Cures Act [79]. In this environment, hospitals often rely on MOTs, even though their standardized architecture restricts adjustments to functions, interfaces, or data flows. Limited involvement of end users during early procurement also contributes to systems that later need upgrades or expensive adjustments, as seen with the electronic health record in Germany [80-82]. These dynamics create fertile ground for organization-technology misfits as described by Strong and Volkoff [19]. Such misfits manifest in fragmented tools, disrupted workflows, higher error risk, and increased resource demands for both clinicians and IT teams [83]. In this study, multiple misfits are reflected in, and help explain, the tensions observed.

Tension 1 is characterized by functionality misfits, data misfits, and usability misfits when systems fail to reflect local workflows or information needs. Consistent with prior research, complex authentication [84], complicated user interface navigation [26], misalignment between systems and workflows [29,85], and limited interoperability [86] contributed to usability, data, and functionality misfits. Together, these factors limited the perceived usefulness of the technologies and thus discouraged adoption while also creating workarounds that made the misfits persistent [29,32,33,87,88]. Structural challenges, such as unreliable Wi-Fi, lack of functional devices [31,84], and difficulties complying with interoperability standards, reinforced these limitations.

Tension 2 reflected role and control misfits. Organizational complexity, unclear responsibilities, and inconsistent oversight made structured participation difficult [35,89]. These patterns mirror earlier studies showing that rigid governance structures and unclear responsibilities impede participation [19,90,91]. In our data, clinicians valued early involvement, whereas project managers emphasized clear contact points and department champions.

Tension 3 reflected role and organizational culture misfits. Clinicians were motivated but lacked time, recognition, and resources to take on implementation responsibilities. Resource constraints limited dedicated work time for both clinicians and project managers, a pattern consistent with the comparatively low IT investment levels in German hospitals [92]. Prior evidence supports that high workload and competing priorities force clinicians to prioritize patient care, limiting their capacity to meaningfully evaluate and adopt MOTs [93-95].

Taken together, these misfit patterns allow a focused reflection on the 3 assumptions from the “Introduction” section. The first assumption, that MOT implementation is shaped by functional and usability misfits, is confirmed but should be broadened. Functionality and usability mattered, but their effects were strengthened by role, control, and cultural misfits, showing that challenges arise from clusters of misfits rather than from product issues alone. The second assumption, that MOT implementation requires capacity-building conditions, is supported but needs refinement. Training, participation opportunities, and protected time are not optional but essential to counteract misfits caused by the limited modifiability of MOTs. However, capacity building alone cannot address structural or cultural misfits when organizations lack available individuals or coordination mechanisms. The third assumption, that structural characteristics intensify misfits, is also supported. Structural conditions not only amplified existing misfits; they also created new roles and cultural misfits by limiting participation, creating inconsistent responsibilities, and reducing available time. Overall, successful MOT implementation requires managing technological, organizational, and cultural misfits together, not only improving product alignment or increasing training.

Based on these findings, we introduce the concept of user:ability. User:ability describes the organizational conditions that enable users to adapt to MOTs effectively. It includes targeted training to clarify relative advantage, structured participation supported by clear communication, and protected time that allows users to engage in implementation tasks. Unlike related concepts such as user participation and user involvement, user:ability does not rely on early co-design, which is usually not possible with MOTs. Instead, it focuses on empowering users during implementation. Strengthening user:ability can help address the 3 tensions by improving perceived relative advantage (resolving tension 1), enabling participation in complex settings (resolving tension 2), and sustaining engagement despite limited resources (resolving tension 3). To operationalize user:ability, the following 3 strategies describe mechanisms to mitigate the tensions identified above, drawing on the facilitators observed in this study and integrating prior evidence.

### Strategy 1: Ensuring Relative Advantage Through IT Integration, Product Alignment, and Targeted Learning

Strategy 1 focuses on reducing functionality, data, and usability misfits by strengthening the perceived relative advantage of the technologies through product alignment, IT integration, and targeted learning.

In this study, relative advantage and compatibility emerged as decisive factors for technology adoption, consistent with diffusion of innovations theory and the Consolidated Framework for Implementation Research [21,36,40]. Tailored learning approaches, including training, local implementation support, process evaluation, and microlearning formats, such as short instructional videos and question-and-answer sessions, helped clarify the benefits of MOT adoption and strengthened user confidence and acceptance [31,85,87,96-103]. As MOTs often require repeated upgrades and costly customizations even after rollout, organizations should integrate MOTs into the IT ecosystem with sufficient in-house IT expertise, enabling real-time access and workflow alignment [104].

### Strategy 2: Empowering User Participation Through Digital Transformation and Structured Communication

Strategy 2 focuses on reducing role and control misfits by creating structures that enable meaningful participation through clear responsibilities, coordination mechanisms, and consistent communication.

Facilitators such as interprofessional networking and collaboration, clear points of contact, and preferred communication channels promoted knowledge-sharing, joint problem-solving, and shared ownership [31,97,100,101,103]. To strengthen these facilitators, hospitals can align IT and organizational goals through a digital business strategy and improve decision-making and coordination through a participatory process model that promotes aligned leadership, adaptive culture, and effective communication [105,106].

Structured and multimodal communication, including intranet resources, structured training, hybrid meetings, and targeted phone calls, can provide tailored support, clarification, and feedback [107]. These measures help align diverse stakeholders, foster trust, and enable coordination across units, thereby reducing role and control misfits.

### Strategy 3: Enabling Clinician Engagement Through Resource Allocation and Systematic Implementation

Strategy 3 focuses on reducing role and cultural misfits by ensuring that individual enthusiasm can translate into sustained engagement through adequate time, support, and recognition.

Hospitals can address these constraints by allocating dedicated resources; providing clear points of contact for clinicians, project teams, and IT support; and structurally embedding clinicians across all phases of implementation [7]. By establishing structured roles such as clinical champions with protected time, for example, 4 hours per week [108], and clear cross-functional points of contact, organizations can sustain engagement, foster ownership, and enhance job satisfaction, supporting effective MOT adoption and evaluation [109-111].

This study contributes to implementation science by moving beyond the common focus on isolated barriers and facilitators. Instead of producing another descriptive list, the analysis integrates the findings into overarching misfits and organizational tensions. This perspective highlights how implementation challenges emerge from structural and relational dynamics rather than from single determinants. The study also introduces the concept of user:ability as an additional contribution. User:ability offers a way to understand how organizations can empower users in settings where technologies allow little adaptation. It shifts the focus from usability to organizational enablement and shows how targeted learning, structured participation, and protected time can help resolve core tensions. This conceptual addition extends existing implementation models by emphasizing the active role organizations play in enabling users to work effectively with MOTs.

While this study focused on barriers to implementing MOTs, the findings should not be interpreted as favoring in-house software development. In-house solutions can involve high development and maintenance costs, limited scalability, and long-term dependence on internal technical expertise and resource commitments [112]. By contrast, off-the-shelf systems benefit from vendor support, regulatory readiness, and standardized functionality, yet implementing them in complex clinical environments frequently requires extensive organizational adaptation to achieve local fit [22,113]. The findings, therefore, do not suggest the superiority of one technological strategy over the other but highlight the need for robust, participatory implementation processes and organizational alignment so that any technology, commercial or locally developed, can be meaningfully integrated into clinical practice.

### Limitations

This study has several limitations that may influence interpretation and transferability. First, researchers held dual roles as implementers and evaluators, which is common in action research but may introduce observer bias. This was mitigated through reflexive practices, independent coding, and triangulation. Second, the analysis relies on frequency-based thresholds to structure qualitative data. Although the thresholds were transparently documented and grounded in established guidance for managing data overload, they may restrict the visibility of less frequent but potentially meaningful insights. As the analysis was conducted at the level of researcher protocols, the findings do not allow conclusions about participant-level prevalence or individual differences.

No demographic data were collected, which limits conclusions about individual differences. While this is methodologically acceptable in action research, where the focus is on collective learning and context-specific change [43], it constrains the ability to examine how individual characteristics may have shaped experiences. The findings, therefore, reflect shared group perspectives within the specific organizational context. Participation was voluntary and may have attracted digitally engaged staff. However, even digitally motivated participants reported substantial barriers, suggesting that the identified tensions reflect systemic conditions rather than the views of a single subgroup. Still, less confident users may be underrepresented.

Some data relied on retrospective accounts, and the short data collection period limited insights into long-term adoption. Moreover, the focus on ready-made solutions may not fully capture upstream influences such as procurement procedures or funding structures. One included technology originated as a local initiative, which only partly aligns with the definition of MOTs but highlights the role of early user involvement.

The study did not include patient-facing technologies and did not collect patient perspectives, limiting insights into how digital transformation affects the care experience. Finally, the study took place in a single, highly resourced university hospital. Transferability is therefore limited, especially to smaller or less digitalized hospitals. Nonetheless, several observed tensions likely resonate across large and complex health care organizations. Future studies should examine these dynamics across hospitals with different structures and digital maturity levels.

### Conclusions

This study demonstrates that, in the context of a German university hospital, the successful implementation of MOTs in hospitals depends less on improving usability and more on strengthening user:ability. This reframes digital transformation as an organizational challenge that requires structures that support learning, participation, and engagement when technologies cannot be adapted to local needs.

The 3 strategies derived from the findings provide context-specific guidance for creating such supportive conditions. Their relevance may extend to other large and complex health care institutions, although the implications should be interpreted in light of the single-site setting and the KHZG-funded implementation environment.

More broadly, hospitals must invest in conditions that enable users to work effectively with digital tools. This includes visible benefits for clinicians, coordinated implementation processes for project teams, and strategic resource allocation from hospital leadership.

These insights also extend beyond MOTs. Any digital technology requires alignment between structures, roles, and resources, so the choice between in-house or off-the-shelf solutions is secondary to an organization’s capacity to implement them effectively. Future research should examine how user:ability can be supported across diverse hospital settings to strengthen digital transformation efforts.

## Data Availability

The datasets generated and analyzed during this study are not publicly available because of data privacy; however, they are available from the corresponding author (LM) upon reasonable request. The qualitative data (field notes, workshop summaries, and focus group materials) generated and analyzed during this study are not publicly available because they contain sensitive information that could compromise participant confidentiality and institutional privacy. This paper includes selected anonymized participant quotations (anchor quotes) that illustrate key themes reported in the findings. No additional data are available for sharing.

## Appendix

Multimedia Appendix 1 Standards for Reporting Qualitative Research (SRQR) checklist.

Multimedia Appendix 2 Consolidated Criteria for Reporting Qualitative Research (COREQ) checklist.

Multimedia Appendix 3 Units of study.

Multimedia Appendix 4 Observation guide.

Multimedia Appendix 5 Table of code frequencies.

Multimedia Appendix 6 Codebook.

## Abbreviations

COREQ: Consolidated Criteria for Reporting Qualitative Research
DHT: digital health technology
GDPR: General Data Protection Regulation
KHZG: Krankenhauszukunftsgesetz (Hospital Future Act)
MOT: modifiable off-the-shelf technology
P: protocol
SRQR: Standards for Reporting Qualitative Research
