# Supplementary material for: Organizational Tensions in the Implementation of Modifiable Off-the-Shelf Technologies in a University Hospital: Qualitative Multimethod Study
Source: JMIR Hum Factors. 2026 May 13;13:e84841. doi: 10.2196/84841 (PMC13216760; doi:10.2196/84841)
Supplement: Multimedia Appendix 1 [file humanfactors_v13i1e84841_app1.docx]

## Qualitative Research Reporting Standards (SRQR) – Checklist

| **No.** | **Topic** | **Item** | **Answer** |
| --- | --- | --- | --- |
| **Title and abstract** |  |  |  |
| S1 | Title | Concise description of the nature and topic of the study identifying the study as qualitative or indicating the approach (e.g., ethnography, grounded theory) and/or data collection methods (e.g., interview, focus group) is recommended | Title clearly states the topic and identifies the study as qualitative multimethod approach |
| S2 | Abstract | Summary of key elements of the study using the abstract format of the intended publication; typically includes background, purpose, methods, results, and conclusions | Structured abstract includes background, objective, methods (qualitative design, data collection, analysis), key results (MOT facilitators and barriers, three tensions), and conclusions. |
| **Introduction** |  |  |  |
| S3 | Problem formulation | Description and significance of the problem/phenomenon studied; review of relevant theory and empirical work; problem statement | The study addresses challenges in implementing modifiable off-the-shelf technologies (MOTs), that offer rapid deployment, but limited adaptability. This is outlined through reviewing relevant literature on digital health, implementation science, and previous empirical work. Problem statement is clearly outlined in the Introduction section |
| S4 | Purpose or research question | Purpose of the study and specific objectives or questions | Examine MOT implementation barriers and facilitators as well as underlying organizational dynamics as outlined in the primary research: “What barriers and facilitators shape the implementation of modifiable off-the-shelf technologies (MOTs), and what organizational dynamics emerge in this process within complex hospital contexts? |
| **Methods** |  |  |  |
| S5 | Qualitative approach and research paradigm | Qualitative approach (e.g., ethnography, grounded theory, case study, phenomenology, narrative research) and guiding theory if appropriate; identifying the research paradigm (e.g., postpositivist, constructivist/interpretivist) is also recommended | The study used qualitative, multimethod collaborative participatory action research design, within a constructivist–interpretivist paradigm,collecting multiple qualitative data sources (observations, workshops, focus groups) to understand how stakeholders experience and act on barriers and facilitators to MOT implementation. (see Study Design;Assessments) |
| S6 | Researcher characteristics and reflexivity | Researchers’ characteristics that may influence the research, including personal attributes, qualifications/experience, relationship with participants, assumptions, and/or presuppositions; potential or actual interaction between researchers’ characteristics and the research questions, approach, methods, results, and/or transferability | The research team consisted of the members of the implementation initiative, including implementation scientists acting as action researchers alongside operational project managers from the hospital’s Chief Medical Information Office (CMIO) and project management unit, acting as client system. Reflexivity was supported through systematic field notes, joint data collection, and regular team discussions, and analytic distance was strengthened by involving two researchers (BF and DF) who had not participated in data collection in leading the analysis. (see Study Design) |
| S7 | Context | Setting/site and salient contextual factors; rationale† | The study was conducted at Charité – Universitätsmedizin Berlin, a large German university hospital (see Setting) |
| S8 | Sampling strategy | How and why research participants, documents, or events were selected; criteria for deciding when no further sampling was necessary (e.g., sampling saturation); rationale† | A multi-stage purposeful sampling (maximum variation, emergent, and criterion-i) captured diverse clinical and managerial perspectives; Breadth and representativeness across roles and departments were prioritized over saturation, given the multimethod, organizational scope of the research; therefore, sampling was not guided by saturation criteria.(see Study Size; Participants) |
| S9 | Ethical issues pertaining to human subjects | Documentation of approval by an appropriate ethics review board and participant consent, or explanation for lack thereof; other confidentiality and data security issues | The study received ethical approval. No identifying data was recorded, and participation was voluntary and uncompensated. Data was securely stored on password-protected servers. (See Ethical Considerations) |
| S10 | Data collection methods | Types of data collected; details of data collection procedures including start and stop dates, iterative process, triangulation of sources/methods, and modification of procedures in response to evolving study findings; rationale† | Data were gathered from clinical staff of different wards and departments, the members of the implementation initiative and project managers in two distinct phases between February and April 2025. (see Data Sources; Setting) |
| S11 | Data collection instruments and technologies | Description of instruments (e.g., interview guides) and devices (e.g., audio recorders) used for data collection; if/how the instruments changed over the course of the study | Observation protocols were developed to document interactions, workflows, and contextual factors during on-site visits, workshops, and focus groups. Field notes were compiled allowing methods and prompts to develop iteratively in response to ongoing field engagement and participants’ perspectives. Data collection relied on manual notetaking without audio or video recording. (See Data Sources) |
| S12 | Units of study | Number and relevant characteristics of participants, documents, or events included in the study; level of participation (could be reported in results) | In Phase 1, 39 clinicians from four wards participated (32 in planning meetings, 7 in a backcasting workshop). In Phase 2, 34 clinicians contributed (23 in focus groups, 11 in feedback meetings), alongside 10 internal team members/researchers. In total, the study involved 129 participants (71 clinicians, 46 project managers, 10 initiative members) and 12 data collection protocols across workshops, focus groups, and observations. (See Data Sources; Setting; Participants) |
| S13 | Data processing | Methods for processing data prior to and during analysis, including transcription, data entry, data management and security, verification of data integrity, data coding, and anonymization/deidentification of excerpts | All data were documented through manual note-taking without audio or video recordings. Notes were digitized, stored on secure password-protected servers, and cross-checked by multiple researchers. Only de-identified data were used for analysis, which was conducted in MAXQDA, with personal identifiers removed to ensure anonymization. (see Data Sources; Ethical Considerations) |
| S14 | Data analysis | Process by which inferences, themes, etc. were identified and developed, including the researchers involved in data analysis; usually references a specific paradigm or approach; rationale† | Data were analyzed using Qualitative Content Analysis (Kuckartz) within MAXQDA. Categories were deductively- inductively developed, partly aligned with CFIR, and organized into second- and first-order categories The process was iterative, team-based, and refined to ensure analytic rigor and contextual relevance(see Data Analysis) |
| S15 | Techniques to enhance trustworthiness | Techniques to enhance trustworthiness and credibility of data analysis (e.g., member checking, audit trail, triangulation); rationale† | Trustworthiness was enhanced through data triangulation (field notes, focus groups, workshops), investigator triangulation (researchers with clinical and implementation backgrounds), and iterative team discussions. An audit trail of coding decisions was maintained in MAXQDA. No member checking was conducted. (see Data Analysis) |
| **Results/findings** |  |  |  |
| S16 | Synthesis and interpretation | Main findings (e.g., interpretations, inferences, and themes); might include development of a theory or model, or integration with prior research or theory | Results are presented in two sections: (1) six integrated second-order categories combining barriers and facilitators within shared aspects of MOT implementation and (2) organizational dynamics conceptualized as tensions.  Barriers reflected Product Limitations (22.6%, 88/390), Misaligned Implementation Process (4.4%, 17/390), Absence of Available Individuals (3.8%, 15/390), Structural Challenges (2.8%, 11/390), and Resource Constraints (2.8%, 11/390). Facilitators included codes around Orchestrated Implementation Process (13.8%, 54/390), Product Alignment (14.4%, 56/390), Effective Coordination and Communication (9.2%, 36/390), Presence of Available Individuals (6.7%, 26/390), Available Resources (3.1%, 12/390), and Structural Assets (1.8%, 7/390). Tensions include generic system vs. Local needs (Tension 1), organizational complexity vs. Structured participation (Tension 2), limited resources vs. Individual enthusiasm (Tension 3) (see Results) |
| S17 | Links to empirical data | Evidence (e.g., quotes, field notes, text excerpts, photographs) to substantiate analytic findings | Data derived from 390 coded segments (172 barriers, 218 facilitators) from clinician and project manager interviews. Categories and tensions were inductively developed from participant perceptions and interpreted by researchers. Direct quotes illustrate barriers and facilitators, e.g., issues with login times, IT infrastructure, lack of training, and engagement: “[...] by the time you've logged in, 3–5 minutes have passed and the others are already with the next patient.” (P9) (see Data Sources; Analysis; Results) |
| **Discussion** |  |  |  |
| S18 | Integration with prior work, implications, transferability, and contribution(s) to the field | Short summary of main findings; explanation of how findings and conclusions connect to, support, elaborate on, or challenge conclusions of earlier scholarship; discussion of scope of application/generalizability; identification of unique contributions to scholarship in a discipline or field | The study identified three tensions in implementing modifiable off-the-shelf technologies: (1) functionality, data, and usability misfits; (2) organizational complexity and unclear responsibilities that hindered participation; and (3) limited time and resources that reduced clinician engagement. These tensions reflect clusters of technological, organizational, and cultural misfits. Based on these findings, the study introduces the concept of user ability and outlines three strategies (1) targeted learning, (2) structured participation, and (3) resource allocation to mitigate these misfits and support effective MOT implementation. (See Discussion) |
| S19 | Limitations | Trustworthiness and limitations of findings | Limitations include the researchers’ dual roles as implementers and evaluators, which may introduce observer bias; this was mitigated through reflexivity, independent coding, and triangulation. Frequency-based thresholds support data reduction but may limit the visibility of less frequent insights. The absence of demographic data restricts conclusions about individual differences, and voluntary participation may underrepresent less digitally engaged users. Retrospective accounts and a short data collection period limit insights into long-term adoption. The study focused on ready-made solutions and may not fully capture upstream influences such as procurement or funding structures. One locally initiated technology only partly aligns with the definition of MOTs. No patient-facing technologies or patient perspectives were included, limiting insights into the care experience. Finally, the single-site, well-resourced setting constrains transferability to other hospital contexts. (see Limitations) |
| **Other** |  |  |  |
| S20 | Conflicts of interest | Potential sources of influence or perceived influence on study conduct and conclusions; how these were managed | All authors stated their individual conflict of interest (see Conflicts of interest) |
| S21 | Funding | Sources of funding and other support; role of funders in data collection, interpretation, and reporting | Funded through KHZG and institutional digitalization budgets; funders had no role in the study or manuscript. (see Funding) |
