# Supplementary material for: Organizational Tensions in the Implementation of Modifiable Off-the-Shelf Technologies in a University Hospital: Qualitative Multimethod Study
Source: JMIR Hum Factors. 2026 May 13;13:e84841. doi: 10.2196/84841 (PMC13216760; doi:10.2196/84841)
Supplement: Multimedia Appendix 2 [file humanfactors_v13i1e84841_app2.docx]

## COREQ: 32-Item Checklist for Reporting Qualitative Studies

| **No.** | **Item** | **Guide questions/description** | **Answer** |
| --- | --- | --- | --- |
| **Domain 1: Research team and reflexivity** | | | |
| **Personal Characteristics** |  |  |  |
| 1. | Interviewer/facilitator | Which author/s conducted the interview or focus group? | C. Gaul, A. Nowak, Lina Mosch, E. Hinz, A. Wels, J. C. Berkmann, C. d. Pasquale |
| 2. | Credentials | What were the researcher's credentials? e.g., PhD, MD | Alessia Nowak (M.Sc.), Carolina Gaul (M.Sc.), Elena Hinz, Julia C. Berkmann (PhD), Cathrin de Pasquale (M.Sc.), Lina Mosch (MD) |
| 3. | Occupation | What was their occupation at the time of the study? | Research Associates in implementation science, members of the project team affiliated with the Chief Medical Information Officer team (CMIO) or project management unit (see Study Design) |
| 4. | Gender | Was the researcher male or female? | All of them are female (see Study Design) |
| 5. | Experience and training | What experience or training did the researcher have? | Operational project managers Chief Medical Information Office (CMIO) or project management unit and implementation researchers from diverse backgrounds including clinical medicine, human factors, and information systems (see Study Design) |
| **Relationship with participants** |  |  |  |
| 6. | Relationship established | Was a relationship established prior to study commencement? | No |
| 7. | Participant knowledge of interviewer | What did the participants know about the researcher? e.g., personal goals, reasons for doing the research | The researchers were introduced as part of the project team and were perceived as collaborators rather than evaluators. Their professional backgrounds and interest in implementation processes were disclosed, while research questions were not shared. (see Study Design) |
| 8. | Interviewer characteristics | What characteristics were reported about the interviewer/facilitator? e.g., bias, assumptions, interests | Some team members were directly involved in coordinating the implementation and facilitating feedback activities, which provided contextual insight but also potential bias. Reflexivity was supported through field notes, joint data collection and analysis, and team discussions, while two separate researchers led the analysis to ensure analytic distance. (see Study Design) |
| **Domain 2: Study design** | | |  |
| **Theoretical framework** |  |  |  |
| 9. | Methodological orientation and theory | What methodological orientation was stated to underpin the study? e.g., grounded theory, ethnography, etc. | Action research: observations, workshops, focus groups (see Study Design) |
| **Participant selection** |  |  |  |
| 10. | Sampling | How were participants selected? e.g., purposive, convenience, snowball | Multistage purposeful sampling strategy  (see Participants) |
| 11. | Method of approach | How were participants approached? e.g., face-to-face, telephone, mail, email | Clinical staff were approached via e-mail ; project manager were approached face to face or via e-mail. (see Study Design) |
| 12. | Sample size | How many participants were in the study? | In Phase 1, 39 clinicians from four wards participated (32 in planning meetings, 7 in a backcasting workshop). In Phase 2, 34 clinicians contributed in focus groups, alongside 10 internal team members/researchers. In addition, 46 project managers were involved (8 in a retrospective meeting, 38 in a workshop). (see Setting; Participants) |
| 13. | Non-participation | How many people refused to participate or dropped out? Reasons? | While participation was generally high, a small number of invited nurses or doctors declined, mostly due to time constraints. Exact numbers of refusals and dropouts were not systematically recorded. (see Participants) |
| **Setting** |  |  |  |
| 14. | Setting of data collection | Where was the data collected? e.g., home, clinic, workplace | Data was collected at the workplace, in a meeting room or online. Workshops with clinicians were held in meeting rooms on the ward, project manager or online. Focus groups were held online or at the workplace. Observations were done at the workplace. (see Data Sources) |
| 15. | Presence of non-participants | Was anyone else present besides the participants and researchers? | No additional non-participating individuals were present. (see Data Sources) |
| 16. | Description of sample | What are the important characteristics of the sample? e.g., demographic data, date | Participants occupations are discloses (e.g. clinician, project manager); Data collection occurred between February and April 2025. (see Participants, Setting) |
| **Data collection** |  |  |  |
| 17. | Interview guide | Were questions, prompts, or guides provided by the authors? Was it pilot tested? | Not Applicable |
| 18. | Repeat interviews | Were repeat interviews carried out? If yes, how many? | Not Applicable |
| 19. | Audio/visual recording | Did the research use audio or visual recording to collect the data? | No audio or video recording was conducted (see Data Sources) |
| 20. | Field notes | Were field notes made during and/or after the interview or focus group? | yes |
| 21. | Duration | What was the duration of the interviews or focus group? | Phase 1:   - Ward visits: 4 observations à 60-90 min - Backcasting workshop: one workshop à 120 min   Phase 2:   - Retrospective focus groups: 4 focus groups à 60 min each - Feedback meeting: one observation à 60 min - Online feedback meeting: one workshop à 90 min - Retrospective workshop: one workshop à 30 min   (see Setting) |
| 22. | Data saturation | Was data saturation discussed? | Given the exploratory nature of the study, data saturation was not systematically assessed. Breadth and representativeness across roles and departments were prioritized over saturation, given the multimethod, organizational scope of the research. (see Study Size) |
| 23. | Transcripts returned | Were transcripts returned to participants for comment and/or correction? | no |
| **Domain 3: Analysis and findings** | | |  |
| **Data analysis** |  |  |  |
| 24. | Number of data coders | How many data coders coded the data? | Three, BK, AN and CG (see Data Analysis) |
| 25. | Description of the coding tree | Did authors provide a description of the coding tree? | Yes |
| 26. | Derivation of themes | Were themes identified in advance or derived from the data? | Themes/Categories were identified using an deductive-inductive approach (see Data Analysis) |
| 27. | Software | What software, if applicable, was used to manage the data? | The qualitative data management software MaxQDA (VERBI GmbH, 2024) was used (see Data Analysis) |
| 28. | Participant checking | Did participants provide feedback on the findings? | No |
| **Reporting** |  |  |  |
| 29. | Quotations presented | Were participant quotations presented to illustrate the themes/findings? Were quotations identified (e.g., ID)? | Yes |
| 30. | Data and findings consistent | Was there consistency between the data presented and the findings? | Yes |
| 31. | Clarity of major themes | Were major themes clearly presented in the findings? | Yes, in First and Second-Order Categories and tensions derived from them (see Data Analysis; Results) |
| 32. | Clarity of minor themes | Is there a description of diverse cases or discussion of minor themes? | Yes. Minor themes are described, as the second-order categories (major themes) are presented together with the codes that constitute them (minor themes).(see Results) |
