# Supplementary material for: Organizational Tensions in the Implementation of Modifiable Off-the-Shelf Technologies in a University Hospital: Qualitative Multimethod Study
Source: JMIR Hum Factors. 2026 May 13;13:e84841. doi: 10.2196/84841 (PMC13216760; doi:10.2196/84841)
Supplement: Multimedia Appendix 3 [file humanfactors_v13i1e84841_app3.docx]

This is a Multimedia Appendix to a full manuscript published in the J Med Internet Res. For full copyright and citation information see http://dx.doi.org/10.2196/jmir.xxxx

**Units of Study**

| Protocol | Type of Meeting | Number of Participants | Profession of Participants | Data Collection Method |
| --- | --- | --- | --- | --- |
| P1 | Retrospective Focus Group | 3 | Clinicians | Focus Group |
| P2 | Retrospective Meeting | 8 | Project managers | Observation |
| P3 | Retrospective Focus Group | 10 | Clinicians | Focus Group |
| P4 | Back-casting workshop | 7 | Clinicians | Workshop |
| P5 | Retrospective Focus Group | 8 | Clinicians | Focus Group |
| P6 | Retrospective Workshop | 38 | Project managers | Workshop |
| P7 | Internal Retrospective Workshop | 10 | Members of the Implementation Initiative | Workshop |
| P8 | Retrospective Focus Group | 13 | Clinicians | Focus Group |
| P9 | Planning Meeting: Kick-off | 8 | Clinicians | Observation |
| P10 | Planning Meeting: Kick-off | 8 | Clinicians | Observation |
| P11 | Planning Meeting: Kick-off | 8 | Clinicians | Observation |
| P12 | Planning Meeting: Kick-off | 8 | Clinicians | Observation |
