# Supplementary material for: Organizational Tensions in the Implementation of Modifiable Off-the-Shelf Technologies in a University Hospital: Qualitative Multimethod Study
Source: JMIR Hum Factors. 2026 May 13;13:e84841. doi: 10.2196/84841 (PMC13216760; doi:10.2196/84841)
Supplement: Multimedia Appendix 4 [file humanfactors_v13i1e84841_app4.docx]

This is a Multimedia Appendix to a full manuscript published in the J Med Internet Res. For full copyright and citation information see http://dx.doi.org/10.2196/jmir.xxxx

**Observation Guide for Preliminary Meetings**

**Guiding Questions:**

1. **IT Infrastructure**

- What technical infrastructure/devices are available? How is the network coverage?
- What are the individual devices used for?
- Are there bottlenecks/disruptions/problems?

1. **Non-verbal Communication**

- Reaction to the implementation initiative (individual phases and steps)
  - What do you think of a participatory implementation approach? (e.g., workshops, interviews, time?)
  - What would you be interested in contributing to?
  - Can staff resources be allocated for participatory implementation?
- Work climate (hierarchical/collegial)
- Openness toward technology

1. **Communication on the Ward**

- How is internal communication organized on site? (Teams channel? Emails?)
- Where/How do you share information such as the introduction of a new technology?

1. **Barriers and Facilitators to Technology Implementation and Use**

- Which digital tools are already being used?
- How were they introduced? What did you particularly like/miss/dislike about it?
- What was the motivation/attitude of staff towards using the technology? Were certain people especially positive/negative?
- What is the attitude of management (clinic directors, senior physicians, nursing management, CCIT experts)?
- Are there designated contact persons?

| **Facilitators** | **Barriers** |
| --- | --- |
|  |  |
|  |  |
|  |  |
|  |  |
|  |  |
|  |  |
|  |  |
