# Supplementary material for: Organizational Tensions in the Implementation of Modifiable Off-the-Shelf Technologies in a University Hospital: Qualitative Multimethod Study
Source: JMIR Hum Factors. 2026 May 13;13:e84841. doi: 10.2196/84841 (PMC13216760; doi:10.2196/84841)
Supplement: Multimedia Appendix 5 [file humanfactors_v13i1e84841_app5.docx]

This is a Multimedia Appendix to a full manuscript published in the J Med Internet Res. For full copyright and citation information see http://dx.doi.org/10.2196/jmir.xxxx

**Codes and frequencies**

Frequencies of coded segments across categories for barriers and facilitators to implementing MOTs in a large German university hospital (n=390 coded segments)

| First Order Category | Second Order Category | Frequencies of coded segments (percentage, absolute) |
| --- | --- | --- |
| MOT Implementation Barriers | Product Limitations | 22.6%, (88/390) |
|  | Resource Constraints | 2.8%, (11/390) |
|  | Structural Challenges | 2.8%, (11/390) |
|  | Misaligned Implementation Process | 4.4%, (17/390) |
|  | Absence of Available Individuals | 3.8%, (15/390) |
|  | Total Reported | 36.4%, (142/390) |
|  | Not Reported | 7.7%, (30/390) |
| MOT Implementation Facilitators | Product Alignment | 14.4%, (56/390) |
|  | Available Resources | 3.1%, (12/390) |
|  | Structural Assets | 1.8%, (7/390) |
|  | Orchestrated Implementation Process | 13.8%, (54/390) |
|  | Presence of Available Individuals | 6.7%, (26/390) |
|  | Effective Coordination and Communication | 9.2%, (36/390) |
|  | Total Reported | 48.9%, (191/390) |
|  | Not Reported | 6.7%, (27/390) |

| Code | % of Barriers | % of total coded segments | % of Facilitators | % of total coded segments |
| --- | --- | --- | --- | --- |
| Relative Advantage | 9.3% (16/172) | 4.1% (16/390) | 16.9% (37/218) | 9.5% (37/390) |
| Technology Guidance or Training | 2.3% (4/172) | 1% (4/390) | 11.9% (26/218) | 6.7% (26/390) |
| Compatibility | 11.6% (20/172) | 5.1% (20/390) | 8.7% (19/218) | 4.9% (19/390) |
| Interprofessional Networking and Collaboration | 1.2% (2/172) | 0.5% (2/390) | 7.3% (16/218) | 4.1% (16/390) |
| Implementation Support | 0.6% (1/172) | 0.3% (1/390) | 6% (13/218) | 3.3% (13/390) |
| Preferred Communication Channel | 1.7% (3/172) | 0.8% (3/390) | 5.5% (12/218) | 3.1% (12/390) |
| Participation | 3.5% (6/172) | 1.5% (6/390) | 4.1% (9/218) | 2.3% (9/390) |
| Process Evaluation | 1.2% (2/172) | 0.5% (2/390) | 4.1% (9/218) | 2.3% (9/390) |
| Clear Point of Contact | 1.7% (3/172) | 0.8% (3/390) | 3.7% (8/218) | 2.1% (8/390) |
| Information Technology Infrastructure | 6.4% (11/172) | 2.8% (11/390) | 3.2% (7/218) | 1.8% (7/390) |
| On-site Implementation Resources | 1.7% (3/172) | 0.8% (3/390) | 3.2% (7/218) | 1.8% (7/390) |
| Champions | 2.3% (4/172) | 1% (4/390) | 2.7% (6/218) | 1.5% (6/390) |
| Capability | 1.7% (3/172) | 0.8% (3/390) | 2.7% (6/218) | 1.5% (6/390) |
| Needs Assessment | 0.6% (1/172) | 0.3% (1/390) | 2.7% (6/218) | 1.5% (6/390) |
| Dedicated Work Time for Implementation | 6.4% (11/172) | 2.8% (11/390) | 2.3% (5/218) | 1.3% (5/390) |
| Engagement | 0% (0/172) | 0% (0/390) | 2.3% (5/218) | 1.3% (5/390) |
| Adaptability | 11.1% (19/172) | 4.9% (19/390) | 1.4% (3/218) | 0.8% (3/390) |
| Organizational Culture | 2.9% (5/172) | 1.3% (5/390) | 1.4% (3/218) | 0.8% (3/390) |
| Promotion | 1.2% (2/172) | 0.5% (2/390) | 1.4% (3/218) | 0.8% (3/390) |
| Standardization | 1.2% (2/172) | 0.5% (2/390) | 1.4% (3/218) | 0.8% (3/390) |
| Trialability | 0.6% (1/172) | 0.3% (1/390) | 1.4% (3/218) | 0.8% (3/390) |
| Variety of Communication Channels | 0.6% (1/172) | 0.3% (1/390) | 1.4% (3/218) | 0.8% (3/390) |
| Implementation Lead | 0% (0/172) | 0% (0/390) | 1.4% (3/218) | 0.8% (3/390) |
| Cross-unit Implementation | 2.9% (5/172) | 1.3% (5/390) | 0.9% (2/218) | 0.5% (2/390) |
| Documentation of Technology Implementation | 2.3% (4/172) | 1% (4/390) | 0.9% (2/218) | 0.5% (2/390) |
| Interoperability | 4.7% (8/172) | 2.1% (8/390) | 0.5% (1/218) | 0.3% (1/390) |
| Funding | 0% (0/172) | 0% (0/390) | 0.5% (1/218) | 0.3% (1/390) |
| Complexity | 14.5% (25/172) | 6.4% (25/390) | 0% (0/218) | 0% (0/390) |
| High Level of Executive Responsibility | 2.3% (4/172) | 1% (4/390) | 0% (0/218) | 0% (0/390) |
| Procurement | 1.7% (3/172) | 0.8% (3/390) | 0% (0/218) | 0% (0/390) |
| Feedback Mechanism | 1.2% (2/172) | 0.5% (2/390) | 0% (0/218) | 0% (0/390) |
| IT Staff Resources | 0.6% (1/172) | 0.3% (1/390) | 0% (0/218) | 0% (0/390) |
| **Total** | 100% (172/172) | 44.1% (172/390) | 100% (218/218) | 55.9% (218/390) |

*Percentages are rounded to one decimal place; totals may not equal 100% due to rounding.
