# Supplementary material for: Organizational Tensions in the Implementation of Modifiable Off-the-Shelf Technologies in a University Hospital: Qualitative Multimethod Study
Source: JMIR Hum Factors. 2026 May 13;13:e84841. doi: 10.2196/84841 (PMC13216760; doi:10.2196/84841)
Supplement: Multimedia Appendix 6 [file humanfactors_v13i1e84841_app6.docx]

This is a Multimedia Appendix to a full manuscript published in the J Med Internet Res. For full copyright and citation information see http://dx.doi.org/10.2196/jmir.xxxx

| **Code name** | **Code definition** | **Reference** | **Quote Barrier** | **Quote Facilitator** |
| --- | --- | --- | --- | --- |
| **Second-Order Category: Product Limitations/Product Alignment:** concerns comments regarding the new technology which is being implemented. This includes statements about its different features, such as relative advantage, compatibility, or adaptability. | | | | |
| (Lack of) Interoperability | applies to statements addressing the ability of different digital systems or applications to effectively communicate and exchange data seamlessly – including technical, semantic, or organizational dimensions. | CFIR and NASSS | *“[There is] different software at the ward, at the intensive care unit and in the outpatient clinic. The software lacks interfaces to each other. There were treatment errors because these interfaces are lacking.”* (P9) | *“Ideal scenarios: linking with telephone” (P4)* |
| High Complexity | applies to statements about how complex it is to learn a new digital tool, to use it efficiently, and integrate it into the workflow. This also includes the additional steps and workload resulting from the implementation of a new tool, encompassing issues such as duplicate documentation and efforts for information upkeep. | CFIR | [Mobile Ward Round] *"You go into the room to see the first patient, document everything, then go to the second patient, then the iPad is off, you have to enter a code and unlock it again, then log back into the HIS—by the time you've logged in, 3–5 minutes have passed and the others are already with the next patient. Then you want to look at the MRI image again, but the Wi-Fi cuts out. So, the idea is great, but the application isn't.* (P9). | - |
| (Lack of) Adaptability | segments were assigned to the code "Adaptability" if they reflected the extent to which the intervention can be adapted to the needs of clinicians and tailored to fit into daily work routines, including the software agility. | CFIR | *[Digital Whiteboard for Ward Management]* “*request for integration with radiology” (P9)* | *"Request for speech recognition → license procurement is in progress. 'This will definitely be of interest to everyone." (P11)* |
| (Lack of) Compatibility | comments addressing the alignment of a new intervention with the beliefs, values and goals of clinicians as well as with the workflow and established clinical procedures. | CFIR | *“If a patient wants a pass afterwards, it is very time-consuming, because discharge management has to contact the OR (Operation Room) nurses, and they then have to laboriously search for it in the OR books.”* (P8). | *[KIM: Communication in Medicine] “KIM is particularly relevant, as we frequently see chronically ill patients and there is extensive communication with other clinics.” (P12)* |
| (Unclear) Relative Advantage | refers to statements about advantages of a new intervention in comparison to current practice or other technologies, perceived by individuals involved in the implementation. | CFIR and NASSS | *“Patient safety is at risk when transferring to the general ward.”* (P12). | *“It’s become our favorite tool – it’s really very effective and saves a thousand phone calls.”* *(P9)* |
| (Lack of) Trialability | refers to comments about the possibility of testing a new technology in a pilot phase before the final decision of a full implementation. | CFIR | *“The hope/expectation for the implementation team was that we would demonstrate how the technologies work and what they can do, rather than simply showing a website and providing information.” (P10)* | *“A doctor tried out the tool [Mobile Ward Round] independently during rounds over the weekend and is thrilled.” (P10)* |
| **Second-Order Category: Presence/Absence of Available Individuals:**  comments about the presence or absence of clinical staff with digital skills, willingness to take responsibility, role-model behavior, availability, and cultural conditions influencing participation in digital projects. | | | | |
| Hindering /Promoting Organizational Culture | applies to statements about formal or informal structures of authority and decision-making levels as well as the atmosphere in the clinic during an intervention. | CFIR | *“I don’t feel valued [or seen], even though, on top of giving 150% in my everyday work, I also take care of introducing the digital technology." (P8)* | *“Openness and interest present” (P10)* |
| (Lack of) Participation | participation in implementation can be understood as the extent to which they take part in, or contribute to, the implementation process. Participation can therefore be assessed by examining the concrete tasks, activities, and behaviors that clinicians or their representatives perform during the planning, adaptation, and execution of implementation efforts. | (Barki & Hartwick, 1994) | *“I [nursing] will have to see who’s really interested in that.” (P10)* | *“[…] became aware of [Medication Management] through another ward and then applied as a pilot ward”* (P3) |
| Presence/Absence of Champions | refers to reports concerning clinicians who play an active role in a new intervention, who support the implementation process by integrating new digital solutions into their workflow, using their expertise to positively influence the intervention, assisting other clinicians to involve their colleagues in the process and giving essential feedback regarding the implementation strategies to improve the whole process iteratively. | CFIR | *“Silence when it came to who might want to be a multiplier and/or participate in workshops.” (P10)* | *“What else do we need from the clinics? Clear, consistent contacts who can serve as multipliers”* (P6). |
| (Lack of) Capability | highlights references to clinicians' knowledge about digital solutions and their capability to employ new technologies | CFIR | *“What is an e-prescription, for example? What is the underlying structure? What can it do? How do you use it?" (P9)* | *“Staff have digital competence”* (P3) |
| Engagement | comprises statements about clinicians’ and leaders’ active commitment, initiative, and motivation during participation in digitalization and implementation. | CFIR | - | *“Nursing outpatient management raises ideas within the team*” (P6). |
| **Second-Order Category: Structural Challenges/Assets:** comments about structural characteristics such as procurement processes and infrastructure in the inner setting that facilitate operational processes. | | | | |
| Deficient/Adequate Information Technology Infrastructure | stands for accounts about technological systems available for the implementation which enable tele-communication, digital documentation and storage, as well as the software and hardware necessary for implementing the new technology. | CFIR | *“iPads should be assigned to the ward/outpatient clinic rather than to individual physicians. This raises the question of how the login process can be simplified — for example, whether multiple logins in parallel on a single iPad would be feasible.”* (P3) | *“[ePrescription] – technical infrastructure requirements already in place.” (P12)* |
| (Lack of) Standardization | refers to the use of formal requirements or normative guidance—whether external (e.g., regulations, accreditation standards, professional society guidelines) or internal (e.g., institutional policies, organizational protocols) —to ensure consistent implementation and delivery of an innovation across departments, hospitals, or entire systems. | CFIR | *[Operating Room Solutions (Digital Implant Passport)]” Specific company information in SAP should be preconfigured (there are no legal requirements for companies to store information digitally, ONLY minimum requirements) “(P8)* | *“ePrescription introduced due to obligation and via intranet” (P8)* |
| Slow Procurement | stands for reports about the process of sourcing and purchasing equipment needed for implementation. | CFIR, NASSS | *“Procurement of technical infrastructure unclear or very slow and bureaucratic via procurement platform“(P3)* | - |
| **Second-Order Category: Deficiencies in/Effective Coordination and Communication:** refer to interprofessional networks and clear communication channels that enable information sharing and collaboration, or, when absent or unclear, hinder coordinated implementation. | | | | |
| (Missing) Variety of Communication Channels | depicts reports about the variety of communication channels that are available during the implementation process to clinicians, project managers and implementation researchers | CFIR | *“Email as the only communication tool is not sufficient → a lot of phone calls with the wards for reminders, appointment scheduling, etc. would be necessary” (P2)* | *“Make use of multiple communication channels: LinkedIn, CV, newsletters, consultation hours, and personal contact.” (P6)* |
| (Unclear) Preferred Communication Channels | applies to statements about the existence of communication channels which clinicians prefer and their identification by implementation researchers or project managers. | CFIR | *“No lessons learned from previous rollouts (e.g., telephone communication, whom and how to address)” (P7)* | *“Pre-announcement via emails and calls”* (P6). |
| (Missing) Interprofessional Networking and Collaboration | comments describing the quality, structure, and processes of collaboration and communication between different professional groups during the implementation. It includes interactions between disciplines (e.g., nurses, physicians, IT, project teams) as well as between organizational units (e.g., wards, clinics, departments) | CFIR | *“Did the various professional groups in a ward implement the system together? Separately – at [Digital Whiteboard for Ward Management], the clinic management connected doctors with [project manager] during early morning meetings, and he independently arranged training sessions with nursing staff. “(P8)* | *“Bringing different professional groups together to plan the implementation.” (P6)* |
| (No) Clear Point of Contact | reflects the extent to which individuals involved in the implementation have a clear point of contact in case of questions or problems. | CFIR | *“Time-consuming creation of lists with an overview of wards and contact persons per ward.” (P2)* | *“[The doctor] gave us specific contacts from the medical staff for implementing digital solutions.”* (P3). |
| **Second-Order Category: Resource Constraints/ Available Resources:** refers to resources available for the implementation process, e.g., training opportunities, funds, on-site IT support, or dedicated work time. | | | | |
| (Lack of) Dedicated Work Time for Implementation | refers to statements about compensated work hours during which clinicians are fully or primarily dedicated to implementation processes, enabling them to completely focus on new interventions. | CFIR | *“She does not have enough resources for an autonomous implementation, she does not have time to explain everything to every colleague and to answer questions.”* (P8). | *“[Employees] require the necessary time and space to both understand and implement digital solutions.” (P7)* |
| (Lack of) On-Site Implementation Resources | refers to statements conveying resources available on-site for the intervention, including the implementation support on-site. | CFIR | *“Actually, at least one person from the [implementation initiative] team should be thoroughly familiar with each project in terms of functions, operation, and general conditions; this person should always be on site during the rollout.” (P2)* | *“Intensive on-site support from the project team on each ward for one week with 2–4 people (was praised and proved worthwhile).” (P2)* |
| Lack of IT staff resources | refers to comments regarding the IT staff resources available for an intervention. | CFIR | *“IT resources required on site for implementation, no resources available for independent implementation.” (P8)* | - |
| Funding | captures references to available funds from external entities such as the government facilitating implementation processes as well as implementation efforts. | CFIR | - | *“Various assistants exempted from KHZG funds“(P3)* |
| **Second-Order Category: Misaligned/Orchestrated Implementation Process:** Implementation process depicts accounts regarding strategies and procedures followed during an intervention. | | | | |
| (Lack of) Implementation Support | concerns comments regarding the project team behind the new intervention and implementation process as well as their approachability perceived by clinicians. | CFIR | *“No technical expertise within the Bauchladen team” (P7)* | *“Use existing meeting appointments to introduce the project.” (P6)* |
| (Absence of) Process Evaluation | captures comments concerning an iterative approach to implementation processes as well as evaluation of these processes, which enables continuous reflection and adaptation. | CFIR | *“No feedback from other departments regarding usage and improvements” (P8)* | “*Planned iterative approach from the start — reminding people, asking about further interaction.” (P7)* |
| (Lack of) Cross-Unit Implementation | applies to reports about the simultaneous implementation process across multiple wards of a clinic, particularly for specialties that often must communicate and collaborate, such as neurology and neurosurgery. | CFIR | *“Preferably used throughout the entire hospital and in adjacent areas [(e.g., Digital Whiteboard for Ward Management, Digital Medication Management (inpatient))] also in outpatient departments – unclear whether already available everywhere).” (P3)* | *“There must be a critical number of wards using the tool [Digital Whiteboard for Ward Management].” (P11)* |
| (Absence of) Needs Assessment | used to code statements concerning the identification of implementation needs and the establishment of implementation strategies tailored to meet them. | CFIR | *„What problems arose during implementation / What could the implementation initiative have done better?” (P7)* | “*Implementation preferences were identified*” (P7). |
| Lack of Feedback Mechanism | highlights references to available feedback mechanisms and the potential for adaptation throughout the implementation process. | NASSS | *“It must be possible to communicate challenges to the central digitalization office / to establish contact with them.” (P3)* | - |
| (Insufficient)Documentation in Technology Implementation | encompasses both records on currently adopted technologies and the systematic reflection on past rollout experiences. | CFIR | *“There is no overview of the units using the technologies. Therefore, the respective departments need to be contacted individually.” (P8)* | *“What worked well? ‘Regular coordination meetings and interaction with key users.” (P11)* |
| Lack of High-Level Executive Responsibility | about statements regarding the increased responsibility of clinicians in executive roles during implementation processes. | CFIR | *“Responsibility is heavily concentrated on leadership, particularly on individual managers.” (P8)* | - |
| (Lack of) Technology Guidance or Training | applies to statements about guidance and training opportunities regarding the use of new digital solutions. | CFIR | *[Operating Room Solutions (Digital Implant Passport)] “Training material: A video is available, but it needs to be updated.” (P8)* | *“Could you show how this can be opened?’ – indicating a wish for a demonstration of the solution (here, the drain tab of the Fluid Balance Module).”* (P10) |
| (Lack of) Promotion | applies to statements about promotion done for the intervention or implementation processes, which provides awareness and reach for new available technologies as well as implementation efforts. | CFIR | *„Technologies unknown“ (P7)* | *“Promoting digitization projects” (P7)* |
| Implementation Lead | defines records about clinicians who take on leadership roles in implementing digital solutions within their clinical setting and among their peers. They are either figures of higher authority or possess greater knowledge about the topic but overall are pioneers in implementing new technologies, who motivate and help their colleagues. | CFIR | *-* | *“[Doctor] as CC IT expert key figure” (P3)* |
